# Post-dengue acute disseminated encephalomyelitis: A case report and meta-analysis

**DOI:** 10.1371/journal.pntd.0005715

**Published:** 2017-06-30

**Authors:** Mohamed Gomaa Kamel, Nguyen Tran Nam, Nguyen Huu Bao Han, Abd-Elaziz El-Shabouny, Abd-ElRahman Mohamed Makram, Fatma Abd-Elshahed Abd-Elhay, Tran Ngoc Dang, Nguyen Le Trung Hieu, Vu Thi Que Huong, Trinh Huu Tung, Kenji Hirayama, Nguyen Tien Huy

**Affiliations:** 1 Faculty of Medicine, Minia University, Minia, Egypt; 2 Online research Club (http://www.onlineresearchclub.org/); 3 Department of Infectious Diseases, Children’s Hospital No.2, Ho Chi Minh, Vietnam; 4 Department of Pediatrics, University of Medicine and Pharmacy, Ho Chi Minh, Vietnam; 5 Kasr Al Ainy School of Medicine, Cairo University, Cairo, Egypt; 6 Faculty of Medicine, October 6 University, Cairo, Egypt; 7 Graduate School of Comprehensive Human Sciences, University of Tsukuba, Tsukuba, Japan; 8 Department of Environmental Health, University of Medicine and Pharmacy in Ho Chi Minh, Vietnam; 9 Department of Neurology, University of Medicine and Pharmacy, Ho Chi Minh, Vietnam; 10 Department of Pediatric Neurology, Children’s Hospital No.2, Ho Chi Minh, Vietnam; 11 Department of Immunology and Microbiology, Pasteur Institute, Ho Chi Minh, Vietnam; 12 Department of Immunogenetics, Institute of Tropical Medicine (NEKKEN), Leading Graduate School Program, and Graduate School of Biomedical Sciences, Nagasaki University, 1-12-4 Sakamoto, Nagasaki, Japan; 13 Evidence Based Medicine Research Group & Faculty of Applied Sciences, Ton Duc Thang University, Ho Chi Minh, Vietnam; 14 Department of Clinical Product Development, Institute of Tropical Medicine (NEKKEN), Leading Graduate School Program, and Graduate School of Biomedical Sciences, Nagasaki University, 1-12-4 Sakamoto, Nagasaki, Japan; Beijing Institute of Microbiology and Epidemiology, CHINA

## Abstract

**Background:**

Dengue is one of the most common infectious diseases. The aim of this study was to systematically review acute disseminated encephalomyelitis (ADEM) and to represent a new case.

**Methodology/Principal findings:**

We searched for articles in nine databases for case reports, series or previous reviews reporting ADEM cases in human. We used Fisher’s exact and Mann-Whitney U tests. Classification trees were used to find the predictors of the disease outcomes. We combined findings using fixed- and random-effects models. A 13-year-old girl was admitted to the hospital due to fever. She has a urinary retention. The neurological examinations revealed that she became lethargic and quadriplegic. She had upper limbs weakness and lower limbs complete paraplegia. Her status gradually improved after the treatment. She was nearly intact with the proximal part of her legs had a mild weakness in discharge. The prevalence of ADEM among dengue patients was 0.4% [95% confidence intervals (95% CI) 0.1–2.5%], all neurological disorders among dengue was 2.6% [95% CI 1.8–3.8%], and ADEM among neurological disorders was 6.8% [95% CI 3.4–13%]. The most frequent manifestation of ADEM was altered sensorium/consciousness (58%), seizures and urination problems (35%), vision problems (31%), slurred speech (23%), walk problems (15%) then ataxia (12%). There was a significant difference between cases having complete recovery or bad outcomes in the onset day of neurological manifestations being earlier and in temperature being higher in cases having bad outcomes (p-value < 0.05). This was confirmed by classification trees which included these two variables.

**Conclusions/Significance:**

The prevalence of ADEM among dengue and other dengue-related neurological disorders is not too rare. The high fever of ADEM cases at admission and earlier onset day of neurological manifestations are associated with the bad outcomes.

## Author summary

We presented a 13-year-old girl of ADEM following dengue infection. She was totally alert and had a low grade of fever with no focal neurologic deficits, on admission. We revealed that the prevalence of either ADEM or all neurological disorders among dengue patients was not too rare. Moreover, we found that the most common manifestation of ADEM was altered sensorium/consciousness followed by seizures and urination problems then vision problems. These manifestations should be considered in the diagnosis and management of dengue-infected patients. Also, this requires shedding the light on the total global cases of ADEM from the annual incidence of dengue. The onset of ADEM can be early or late after dengue infection. Hence, clinicians should pay attention that it can be early or late that patients can forget about their fevers. Moreover, the onset day of neurological manifestations and patients’ temperature were significantly associated with the disease outcome.

## Introduction

Dengue, a worldwide prevalent mosquito-borne infectious disease, is a *flavivirus* spread by several species of *Aedes* type mosquitos, mainly *Aedes aegypti* [1]. Dengue has become a dangerous burden and is widely spread in more than 110 countries [2, 3]. The incidence of dengue has increased to reach 30-fold throughout the past 50 years [4]. Annually, between 50 and 528 million people have the infection and about 10,000 to 20,000 deaths [5–8]. Dengue has a wide variety of manifestations, from fever to dengue shock syndrome and/or multiple organs failures [1, 9]. There are a series of biological predictors such as immune cytokines [10–12], circulating DNA [13], microalbuminuria [14], nonstructural protein 1 [15–17], IgM, IgG [18], IgA [19] and endothelial cell damage, as well as dysfunction predictors, have been evaluated [20]. However, no efficient marker for the prediction of severe dengue infection has been discovered [20–22]. Although neurological problems of dengue virus (DENV) have also been reported, the incidence of this group is uncommon between 0.5 and 6.2% [23]. A previous systematic review has investigated the factors associated with DENV and revealed that these factors included the neurological signs [24]. DENV associated neurological problems can be divided into DENV direct invasion and para- or post-infectious disease [3]. These neurological DENV include encephalopathy, encephalitis, immune-mediated syndromes as acute disseminated encephalomyelitis (ADEM) and Guillain-Barré syndrome (GBS), neuromuscular complications as hypokalemic paralysis and dengue-associated stroke [25–30]. ADEM in dengue is very rare and it may occur during the acute phase or post-infectious phase of dengue. It is known to involve an immune-mediated mechanism in which the cytokine overproduction is triggered by DENV [25]. There was another theory which is the immune-mediated attack by autoantibodies and/or T-cells to central nervous system myelin structure. This leads to acute demyelination of the white matter of the brain, spinal cord or both. Thus, it results in an altered mental status and focal neurologic findings in ADEM patient such as paralysis [3]. Although ADEM causes a significant impact on dengue patients, data about this complication is still lacking. Understanding of this complication provides a potential insight into the clinical picture of DENV infection. Thus, this study aimed to conduct an extensive systematic review and meta-analysis of the literature on the ADEM manifestations in dengue with a new case report.

## Methods

### Ethics statement

All the methods were performed in accordance with the relevant approved guidelines, regulations and declaration of Helsinki. The experimental protocols were approved by Children’s Hospital No.2 in Ho Chi Minh City in Vietnam. Written informed consent was obtained from the parents to have their girl’s details and accompanying images published and approved by the aforementioned hospital. Moreover, all patient’s data was analyzed anonymously.

### Search strategy and study selection

This systematic review was performed according to the Preferred Reporting Items for Systematic Review and Meta-analyses statement (PRISMA) (S1 Table) [31]. We had developed and registered a protocol of methods (CRD42016047583). From inception to the 12^th^ of September 2016, we searched for suitable studies in nine databases including; PubMed, Google Scholar, Institute of Science Index (Web of Science), Scopus, Popline, World Health Organization Global Health Library, Virtual Health Library, New York Academy of Medicine Grey Literature Report, System for Information on Grey Literature in Europe and cross-references from the included articles and previous reviews. The search strategy used was (ADEM or encephalomyelitis) and dengue. Three independent reviewers initially scanned primary titles and abstracts (when available) to select potential full-text articles for further scrutiny according to the inclusion and exclusion criteria. The inclusion criteria were as following; case reports, case series, previous literature reviews or systematic reviews discussing post-infectious immune-mediated ADEM of dengue infection in human. Exclusion criteria were as following; other study designs rather than case reports, case series, previous literature reviews or systematic reviews, other complications rather than ADEM, overlapped data sets, data which could not be extracted, duplicated studies and unreliable or incomplete data, no full-text available, abstract-only articles (conference, letters, commentaries), or thesis, books, review editorial or author response. When the title and abstract were not rejected by any reviewer, the full-text of the article was obtained and carefully reviewed for inclusion by the three reviewers. Inclusion or exclusion of each study was determined by discussion and consensus between the three reviewers. When the disagreement occurred, a consensus decision was made following discussion with a senior reviewer.

### Data extraction

Data were extracted by three authors and were checked by at least another author. The disagreement was resolved via discussion and a consensus reached between the three authors. The data extraction form in an Excel file was developed by two authors based on a pilot review and extraction. The data extracted included the first author, year of publication, year of patient recruitment, study design, country of origin and characteristics of the population (infant, children, adult), gender, age at examination of included individuals, the manifestations, the blood and CSF analyses, medications used, visual and neurological examinations, renal and liver function tests and outcome of each patient. If there were more than one value from the examination, the nadir value, e.g. the lowest platelets, the highest packed cell volume (PCV), was extracted. Papers published by the same research group and studying the same factor were checked for potentially duplicated data based on the year of patient recruitment and hospital where the patients were recruited. When duplications were noted, the largest data set was used for our study.

### Statistical analysis

Fisher’s exact and Mann-Whitney U tests were used for categorical and continuous variables, respectively. The values with different units were converted into one common unit to make the values comparable. The classification tree models were used to find the independent predictors that best predict bad outcomes (partial recovery or death) versus complete recovery as well as complete recovery versus partial recovery [32]. In particularly, 20 variables including age, sex, clinical examination’s variables and the steroid treatment and its administration route were included to build classification tree models. We selected the maximum depth of the tree to be five to construct a tree of reasonable complexity. If the tree is too complex, it is difficult to apply. Likewise, we chose the minimum number of observation at each leaf node is equal to five to prevent the tree from sub-dividing into overly specific nodes that contain little supporting data. The performance measures of the tree were accuracy (1 –misclassification error) and its 95% confidence intervals (95% CI), sensitivity, specificity, positive predictive (PPV) and negative predictive values (NPV). The statistical significance was considered when the p-value was < 0.05. Data were analyzed using SPSS version 23.0, and R software version 3.3.2.

### Meta-analysis

Meta-analyses were performed using Comprehensive Meta-analysis (CMA) software version 3 (Biostat, NJ, USA) when there was more than one study. Dichotomous variables were analyzed to compute pooled event rate (ER). A fixed-effect model [33] was used when there is no evidence of a heterogeneity between studies, otherwise, a random-effects model was chosen [34]. Heterogeneity between studies was evaluated using the Q statistic and I^2^ test which describes the percentage of variability in the effect estimates that is because of heterogeneity beyond sampling error [34, 35]. To evaluate the presence of publication bias, we performed Begg’s funnel plot [36] and Egger’s regression test [37, 38] when there were five or more studies in the analysis. The publication bias was considered significant when the p-value was < 0.1. If the publication bias was found, the trim and fill method of Duvall and Tweedie was performed by adding studies that appeared to be missing [39, 40] to enhance the symmetry [41]. The adjusted pooled effect size and its 95% CI were computed after the addition of potential missing studies.

## Results

### Case report

**Fig 1:**
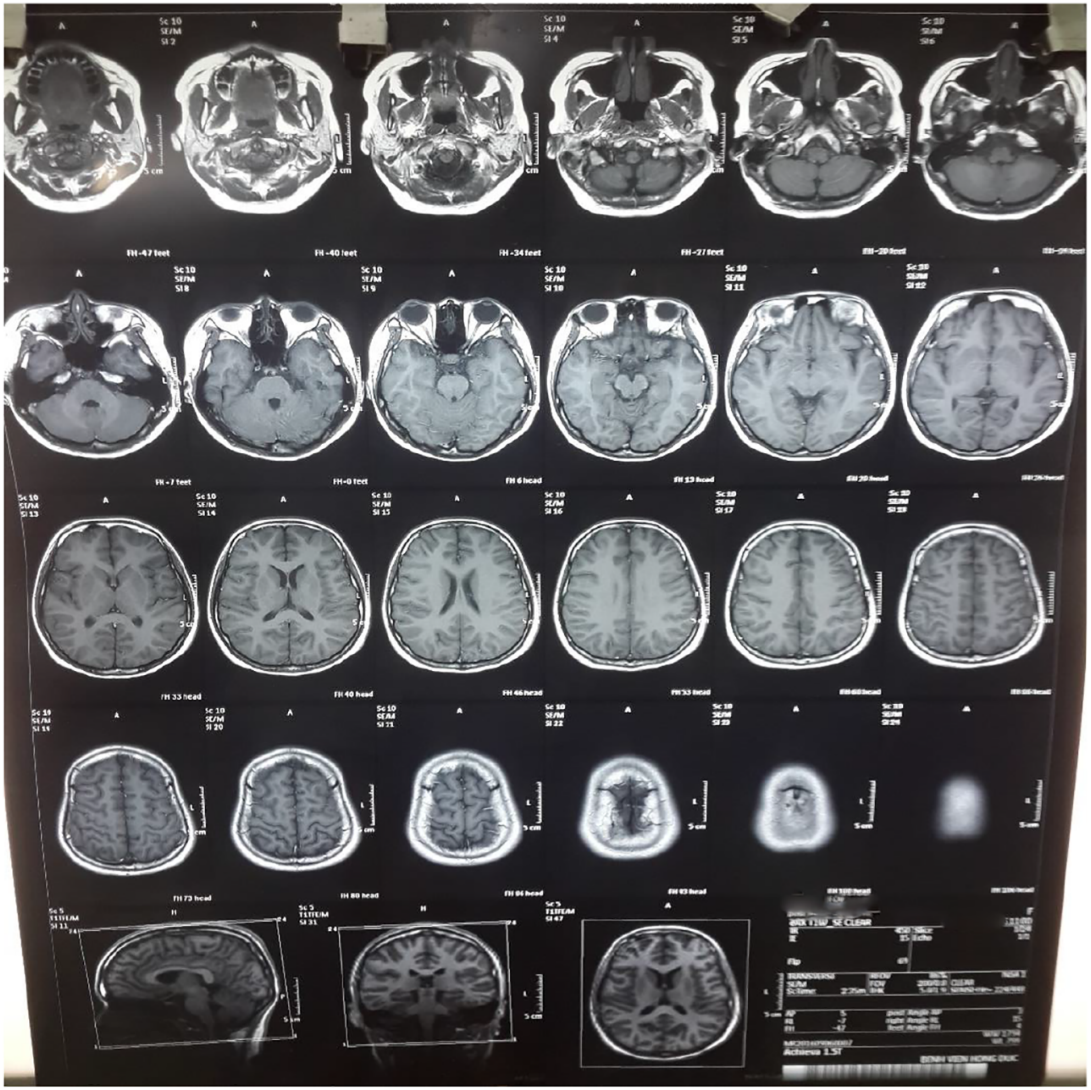
Magnetic resonance imaging (MRI). Axial non-contrast (fluid-attenuated inversion recovery (FLAIR) MRI.

A 13-year-old girl was admitted to Children’s Hospital No.2 in Ho Chi Minh City in Vietnam on 31^st^ August 2016 because of fever for seven days. On admission, she was totally alert and had a low grade of fever. The examination found no focal neurologic deficits. Her total blood count showed that white blood cell (WBC) and platelet counts were 11,000/μL and 182,000/μL, respectively. C-reactive protein level was 3 mg/L. On the 8^th^ day (the day after admission) of her disease course, she recovered from fever but first began to complain of no passage of urine. She was found to lose the sensation of urinating and have urinary retention. She then needed insertion of the indwelling urinary catheter. On the 9^th^ day (the 2^nd^ day of admission), the serologic test revealed that serum dengue IgM was positive. Serum PCR dengue was negative. Laboratory tests of liver and renal functions and electrolytes did not show any particular abnormality. On the same day, she could not move her legs, began to lose her consciousness and showed signs of confusion. The neurological examination then found that she became lethargic and quadriplegic with no abnormal sign of cranial nerves. She had normal ocular fundus, opened her eyes with painful stimulation, answered her name and then drifted back to sleep. Muscle strength and tendon reflexes of the upper extremities were 2/5 and 2+, respectively, with the weakness of upper limbs (UL). The sensory functions were nearly intact. The muscle strength and tendon reflexes of the lower extremities were 0/5 and 2+, respectively, with complete paraplegia of lower limbs (LL). Bilateral (B/L) Hoffmann and Babinski tests were positive. Cerebrospinal fluid (CSF) collected on 10^th^ day showed pleocytosis (61 cells/mL); elevation of protein, 1.68 g/L; glucose, 0.43 g/L; chloride, 138 mmol/L; lactate 4.23, mmol/L. Her CSF was positive for ELISA dengue IgM but negative for ELISA Japanese encephalitis virus IgM. PCR Zika virus in blood and in urine was negative. Magnetic resonance imaging (MRI) of the brain and spinal cord did not show any particular abnormality (Fig 1 and S1–S3 Figs). An electromyogram showed that motor and sensory functions were normal on both UL and LL (S4 and S5 Figs).

From her clinical course and laboratory tests, she was diagnosed as ADEM following dengue infection without warning signs. For treatment of ADEM, high dose of methylprednisolone (30mg/kg/day) for five days was given, beginning on the 3^rd^ of September. The oral low dose of prednisone (1 mg/kg/day) was then used for four weeks. Her alertness improved gradually. On the fourth day of methylprednisolone course, she opened her eyes responding to voice, oriented and answered word by word correctly. Although urinary retention remained, her muscle strength of upper and lower extremities increased to 4/5 and 2/5, respectively. After two weeks of oral prednisolone, limbs weakness was significantly improved and after four weeks, sphincter function was back to normal. She was nearly intact with the proximal part of her legs had a mild weakness when she was discharged from the hospital after four weeks of admission (five weeks since fever onset).

### Search results

**Fig 2:**
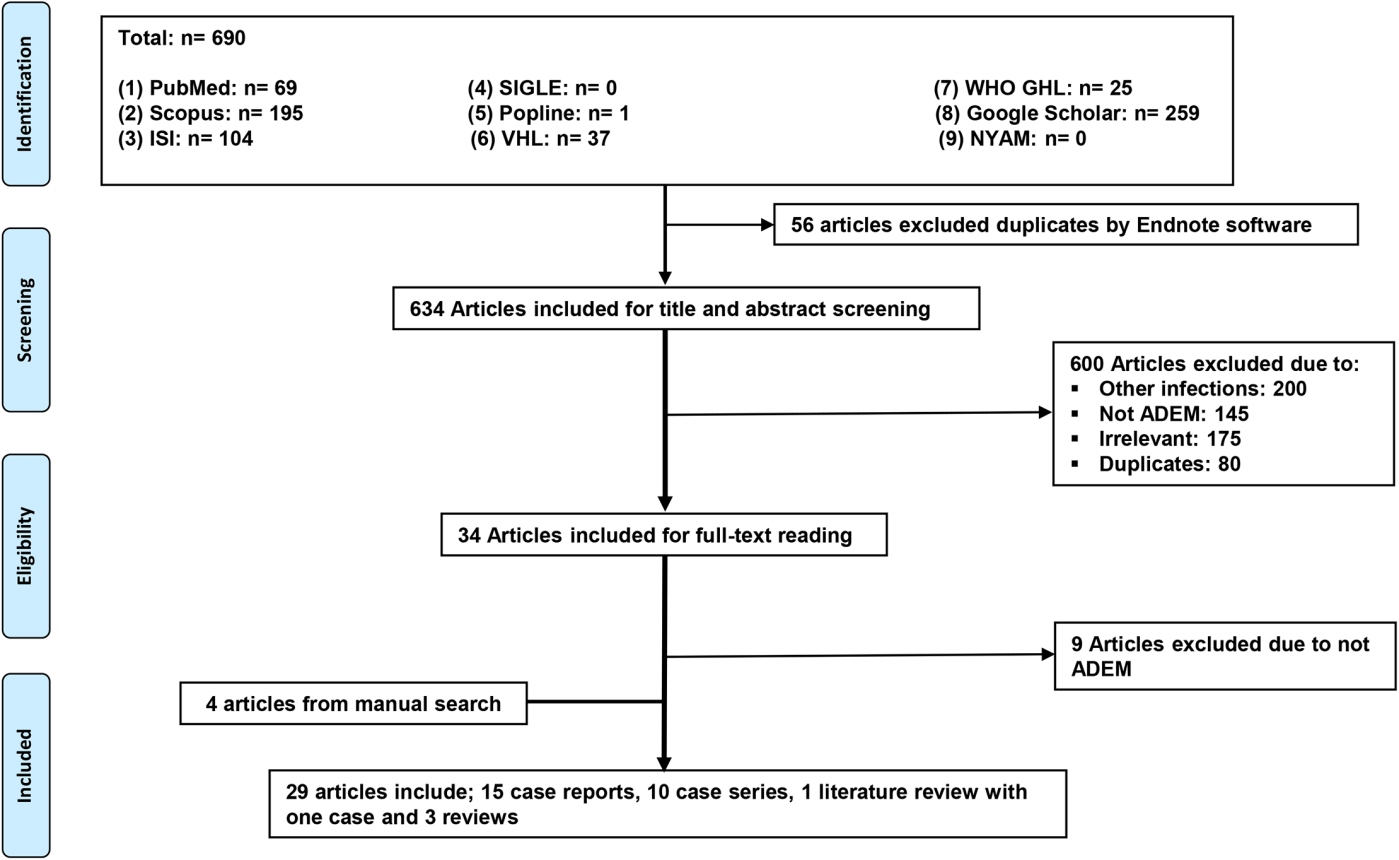
PRISMA chart showing the flow of publications via the review process.

From nine databases, we identified 690 potentially relevant publications. After excluding duplicates and screening titles and abstracts, we retrieved 34 articles for full-text review. Of these, 25 articles met our inclusion criteria. Four additional articles, from manual search, were identified. Finally, 29 articles were in this systematic review including; 15 case reports, 10 case series, 1 literature review with 1 case, and 3 literature reviews (Fig 2).

### Study and patient characteristics

The total sample size was 1,163 dengue patients including 165 patients with neurological complications. Of those 165 patients, there were 29 ADEM cases including three cases of rare types from ADEM; one case with neuromyelitis optica [42], two cases with meningoencephalitis [43, 44], and our new case. The characteristics of the 29 cases are shown in Table 1. Among 29 ADEM patients, there were three cases were described in a group of neurological manifestations with limited individual data [43, 45]. There were more males than females (18 and 8, respectively), the median age was 20 (range 9 days to 65 years). Most of the dengue cases were diagnosed based on IgM followed by IgG.

**Table 1:** Characteristics of the included cases.

Author, year	ADEM No./neuro disorders No./sample size	Age*/sex	Admission reasons	Dengue diagnosis; IgM/IgG/NS1/RT-PCR
**Pal, 2016 [44]**	1/9/9	19/M	Fever, headache, and vomiting	+/ND/ND/ND
**Moura, 2004 [43]**	1/31/31	54/F	ND	ND/ND/ND/ND
**Abdulrazak, 2015 [75]**	1/1/1	9 days/M	Poor feeding and fever	-/+/+/ND
**Sundaram, 2010 [55]**	1/1/1	27/M	Fever, chills, rigors, headache, vomiting and altered sensorium	+/+/+/ND
**Singh, 2015 [76]**	1/1/1	6/M	Walk problems, dribbling of urine and weakness	+/+/+/ND
**Gera, 2010, [77]**	1/1/1	27/M	Fever, myalgia, and vomiting	Unclear/unclear/ND/ND**
**Gupta, 2013 [61]**	1/1/1	26/F	Fever, vomiting, arthralgia and myalgia	+/ND/+/ND
**Yamamoto, 2002 [49]**	1/1/1	58/M	Fever and erythema	+ /+/ND/ND
**Kumar, 2014 [78]**	1/1/1	16/M	Fever, chills and rigor, headache, vomiting, and muscle pain	+/ND/ND/ND
**Brito, 2007 [79]**	1/1/1	37/F	Asthenia, headache, myalgia, and arthralgia	-/ND/ND/ND
**Bhat, 2010 [51]**	1/1/1	14/M	Fever, vomiting, headache, altered sensorium, and seizures	+/ND/ND/ND
**Chakrabarti, 2015 [52]**	1/1/1	29/M	Fever and seizures	+/-/ND/ND
**Cunha-Matta, 2004 [53]**	1/2/2	10/F	Fever	+/ND/ND/ND
**Chowdhury, 2011 [54]**	1/1/1	13/F	Fever and convulsions	+/-/ND/ND
**Gupta, 2015 [68]**	2/2/2	40/M	Urinary retention and walk problems	+/+/-/ND
		25/M	Fever, chills, headache, and vomiting	+/-/ND/ND
**Dewan, 2016 [56]**	1/1/1	17/M	Fever, vomiting and altered sensorium	+/ND/+/ND
**Gala, 2012 [80]**	1/2/2	8/F	Fever, vomiting, abdominal pain, and urinary retention	-/+/ND/ND
**Verma, 2011[81]**	1/26/26	11/M	Fever, headache, and vomiting	+/ND/ND/ND
**de Sousa, 2006 [42]**	1/1/1	11/F	Fever, myalgia, ocular pain, and arthralgia	ND/ND/ND/ND
**Koshy, 2012 [57]**	2/21/799	32/M	Altered sensorium, and hemiparesis	ND/ND/ND/ND
		26/M	Altered sensorium	+/ND/ND/ND
**Karoli, 2016 [82]**	1/1/1	32/F	Fever, chills, myalgia and arthralgia, abdominal pain, and vomiting	+/ND/ND/ND
**Puccioni-Sohler, 2009 [62]**	1/10/10	65/M	Fever, malaise, and thrombocytopenia	+/+/ND/ND
**Pan, 2016 [83]**	1/1/1	15/M	Fever	+/ND/ND/ND
**Fragoso, 2016 [84]**	1/1/1	20/M	Diplopia, optic neuritis, paraparesis, paresthesia, and hyperreflexia	ND/ND/ND/ND
**Ferreira, 2005 [45]**	1/41/41	Unclear**	Unclear**	Unclear**
**Wasay, 2008 [28]**	1/6/255	Unclear**	Unclear**	Unclear**
**Our present case**	1/1/1	13/F	Fever	+/ND/ND/-

Abbreviations; ND = Not Described, No. = Number, NS1 = non-structural protein 1, IV = Intravenous, CR = Case Report, LR = Literature Review, CS = Case Series, + = Positive, - = Negative, M = Male, F = Female, LL = Lower Limb, HIV = Human Immunodeficiency Virus, H = Hepatitis, M = Malaria, CMV = Cytomegalovirus, EBV = Epstein–Barr Virus, HSV = Herpes Simplex Virus, JE = Japanese Encephalitis, MT = Mycobacterium Tuberculosis, HTLV = Human T-Lymphotropic Virus, Ig = Immunoglobulin, RT-PCR = Reverse Transcriptase- Polymerase Chain Reaction.

*Age is in years otherwise stated.

**Unclear means that the information in this paper, which contained more than one case, were not specified for ADEM case only.

The three included literature reviews, in general, discussed the pathogenesis of DENV and its accompanying neurological complications, their pathogenesis, and their incidence. The first review discussed the neuropathogenesis of DENV illness, its neurological complications, the diagnosis, and treatment of these diseases. It discussed also the epidemiology of DENV and the increasing prevalence and incidence of the disease and its extension to new countries. The neuropathogenesis of DENV includes three ways; the metabolic disturbance causing encephalopathy, direct central nervous system invasion (especially, by DENV-2 and -3) causing mainly encephalitis, and autoimmune reaction mechanism. The neurological complications discussed included encephalitis and meningitis, being the most common complication and caused by direct invasion, ADEM, and its rare type neuromyelitis optica, by an immune-mediated process, myelitis, either by an immune-mediated mechanism or by direct invasion, GBS, and mononeuropathies, by autoimmune mechanisms, and myositis [46].

While the second one discussed the various neurological complications, their diagnosis, and the treatment. The neurological complications included dengue encephalopathy, describing it as the most commonly reported neurological disorder associated with DENV and stating that in a retrospective study in Indonesia, 6% (152) of patients with DHF were admitted with encephalopathy. Encephalitis was described in five studies. Post-dengue immune-mediated diseases were discussed and included acute transverse myelitis, GBS, ADEM, and its rare type neuromyelitis optica. Also, cerebrovascular complications (with unknown incidence) and dengue muscle dysfunction (ranging from 66 to 100% in different studies) and neuro-ophthalmic complications (about 10 to 40% in different studies) were described [47].

Finally, the last one discussed the epidemiology, transmission of DENV, its clinical manifestations, its neurological complications and their pathogenesis, the diagnosis, and management of the disease. The pathogenesis of the neurological complications included immune-mediated reactions, metabolic disturbance, and direct invasion. This study described encephalopathy as the most common neurological manifestation of DENV infection. Other neurological complications were encephalitis, myelitis, GBS, myositis, and hypokalemic paralysis. However, ADEM was described as a rare complication [48].

### Meta-analysis results

**Fig 3:**
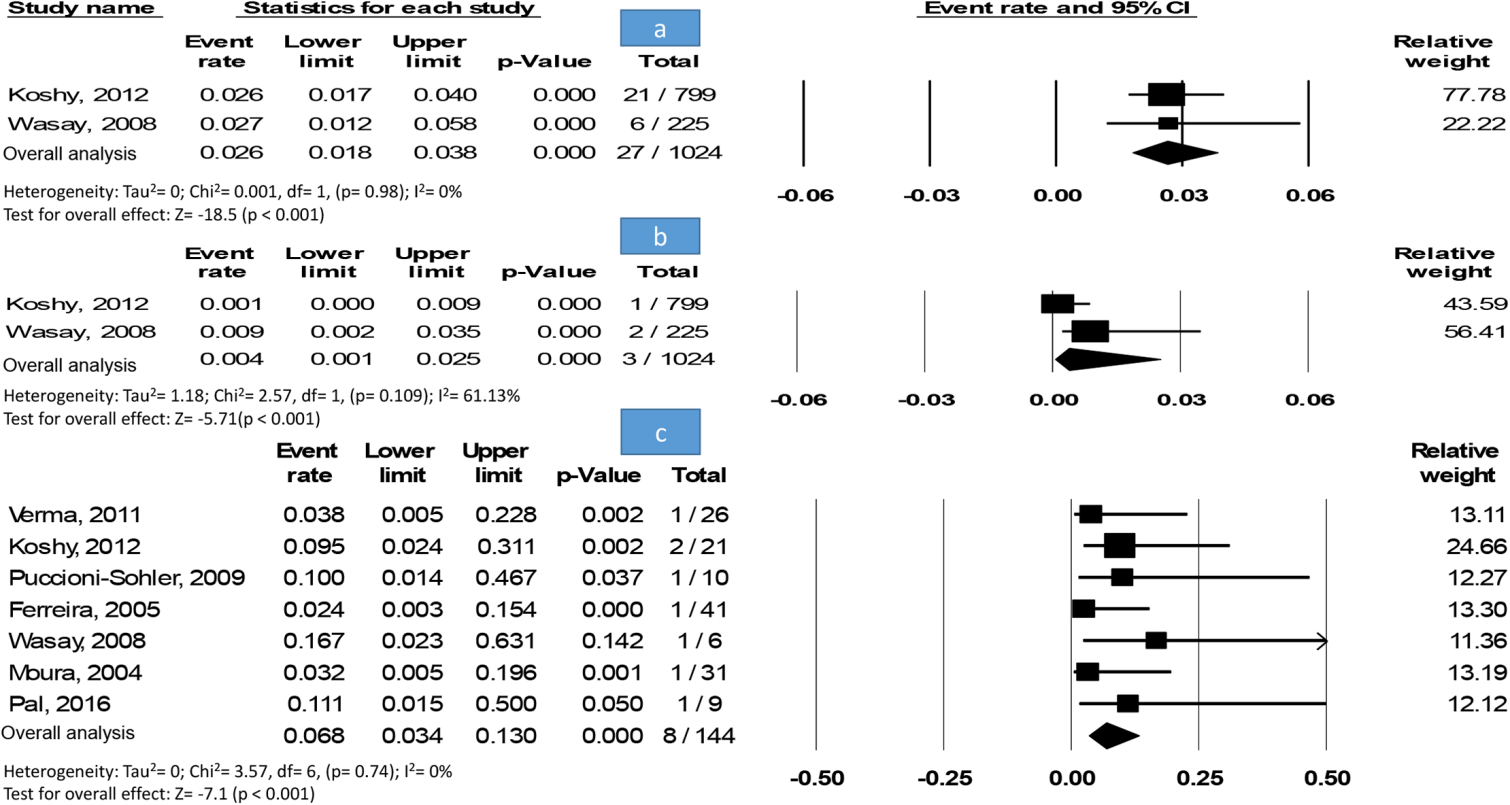
Meta-analysis. (A) Forest plot of ADEM prevalence among other neurological disorders. Showing the pooled event rate with 95% CI using fixed-effect model. (B) Forest plot of ADEM prevalence within dengue patients. Showing the pooled event rate with 95% CI using a random-effects model. (C) Forest plot of neurological disorders prevalence within dengue patients. Showing the pooled event rate with 95% CI using fixed-effect model.

The prevalence of neurological disorders (n = 27) among dengue patients (n = 1,024) in two studies was 2.6% [1.8–3.8%] without an evidence of heterogeneity (Fig 3A).

Pooling two studies enrolling all available dengue patients (n = 1,024) revealed that the prevalence of ADEM (n = 3) among dengue patients was 0.4% [0.1*–*2.5%] with a moderate heterogeneity (Fig 3B).

In seven studies recruiting dengue patients (n = 144) with neurological disorders (n = 8), the prevalence of ADEM was 6.8% [3.4–13%] without an evidence of heterogeneity nor publication bias, Egger’s test (p-value = 0.8) (Fig 3C).

### Manifestations

We could only analyze manifestations in 26 cases due to the lack of information in three cases [43, 45].

The onset day of neurological manifestations after initial dengue symptoms ranged from day 3 to day 19 (median = 7). Most of the cases had a fever on the ADEM onset (25 cases) and 4 cases were not described (ND). The reasons for ADEM admission were fever, vomiting, urination problems, arthralgia, seizures, walk problems, chills, altered sensorium, asthenia, hemiparesis, vision problems, paraparesis, paresthesia and hyperreflexia, abdominal pain, thrombocytopenia, lethargy, poor feeding and seizures, weakness, headache, rigors, and/or myalgia.

**Fig 4:**
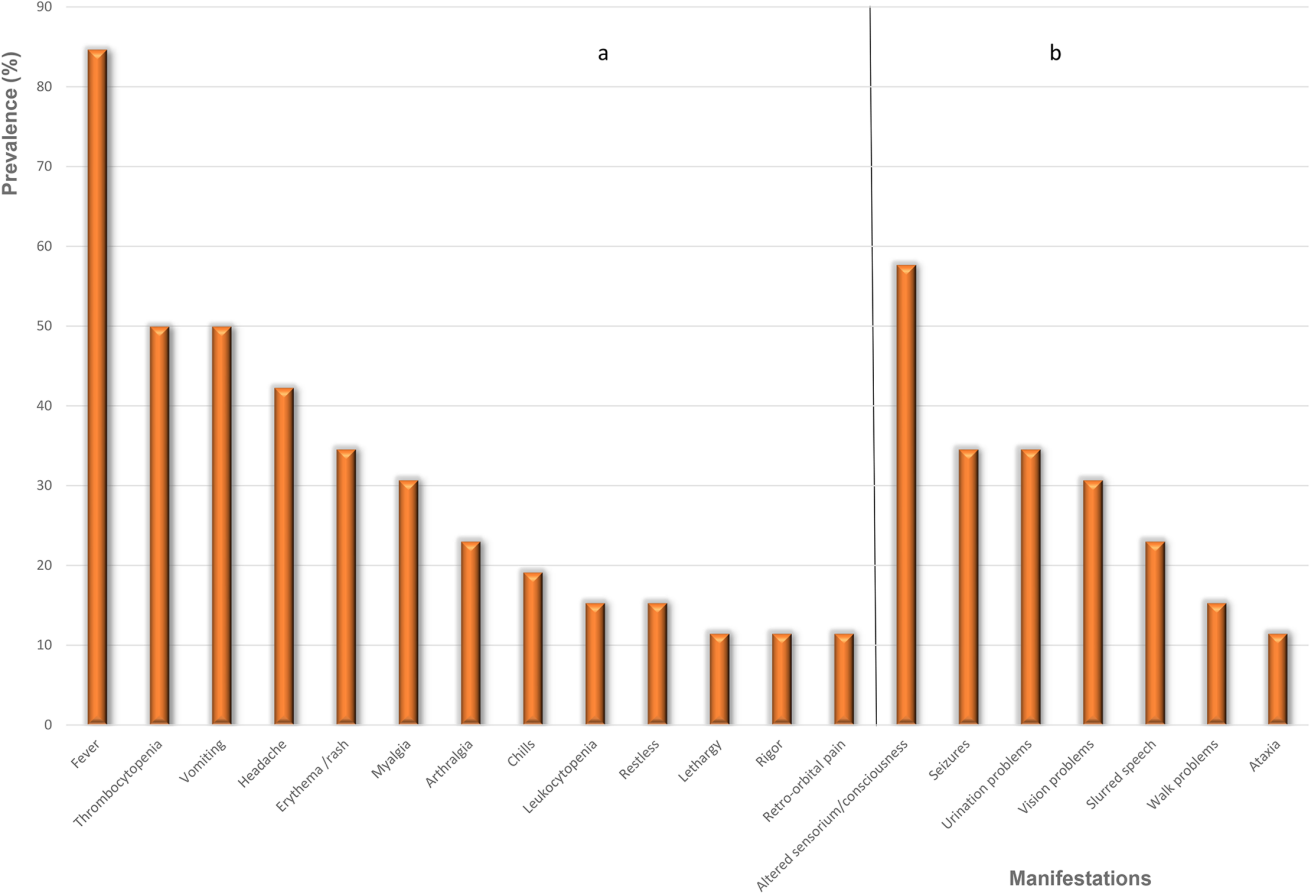
The frequency of each manifestation if included in more than two cases. (A) dengue-related manifestations, (B) ADEM-related manifestations.

The most frequent manifestations and signs related to dengue were fever (22/26, 85%), thrombocytopenia and vomiting (13/26, 50%), headache (11/26, 42%), erythema/rash (9/26, 35%), myalgia (8/26, 31%), arthralgia (6/26, 23%), chills (5/26, 19%), leukocytopenia and restless (4/26, 15%) then retro-orbital pain, rigors and lethargy (3/26, 12%). While the most frequent manifestations and signs related to ADEM were altered sensorium/consciousness (15/26, 58%), seizures and urination problems (9/26, 35%), vision problems (8/26, 31%), slurred speech (6/26, 23%), walk problems (4/26, 15%) then ataxia (3/26, 12%) (Fig 4 and S2 Table).

Liver function tests were normal in 5 cases, abnormal then normal in 1 case, abnormal in 9 cases and ND in 14 cases. The renal function tests were normal in 3 cases, 1 case had urea: 46 mg/dl and creatinine 1.1 mg/dl, 1 case had acute kidney injury and metabolic acidosis and ND in 23 cases. Urinalysis was normal in 3 cases and ND in 26 cases. Chest-X rays were normal in 4 cases, suggestive of acute respiratory distress syndrome (ARDS) in 1 case and B/L fluffy shadows as well as ARDS in another case and ND in 23 cases. The cardiovascular system examination was normal in two cases and ND in 27 cases. The pulses per min were normal in most cases, median (range) was 110 per minute (92–140), stable in 2 cases but ND in 9 cases. The abdominal findings were normal in 1 case, distended urinary bladder, splenomegaly 2 cm and hepatomegaly 3 cm in another case, markedly distended and palpable urinary bladder and spleen tip palpable, 1 case had pain, mild hepatomegaly in another case and ND in 24 cases. The arterial blood pressure (ABP) was normal in most cases, median (range) of systolic blood pressure was 100 mmHg (90–130) while of diastolic blood pressure was 60 mmHg (60–80) and ND in 19 cases. The tourniquet test was positive in 3 cases and ND in 26 cases. The respiratory rate was 28/min in 1 case, 30/min in 1 case and stable in another case and ND in 26 cases (S3 Table).

The results from CSF analysis showed that most of the cases have elevated protein levels (13 cases), normal glucose (11 cases), pleocytosis (6 cases), positive for ELISA dengue IgM in 2 cases and ND in 12 cases. The results from MRI of brain and spinal cord showed that most of the cases have abnormalities such as T2 lesions, demyelination 2 cases and B/L hemorrhagic demyelination in 1 case, cervical/proximal dorsal cord edema deep white matter, cortical and Pontine swellings in 1 case, cervical/proximal dorsal cord edema in 1 case, cervicodorsal cord swelling in another case and no description for MRI of spinal cord in 20 cases while in 7 cases only for MRI of brain (S4 Table).

The power grade ranged from 1/5 to 5/5. Moreover, deep tendon reflexes (DTRs) were normal (3 cases), increased/hyperreflexia (3 cases) or brisk (3 cases), absent/hyporeflexia (2 cases) and ND (18 cases). Plantar responses were B/L extensor (9 cases), B/L Babinski sign (1 case), flexor (1 case), B/L Hoffmann and Babinski signs (1 case), left Babinski sign and absent response in right foot (1 case) nonresponsive B/L (1 case) and ND in 15 cases. Cranial nerves were normal (6 cases), unable to be examined (3 cases) facial nerve palsy (1 case), B/L ptosis (1 case) and ND (18 cases). Furthermore, the ULs and LLs were abnormal in most cases, normal in 1 case only and ND in 11 cases for LLs and cases for ULs 13 cases. The motor system was ND in 25 cases and has abnormalities (hemiparesis or quadriparesis) in the remained ones (S5 Table).

The blood analysis showed an increased WBC in most cases, median (range) of WBC = 45 × 10^8^/L (0.011–31×10^9^), while of platelet was 60 × 10^9^ (1.1 × 109–328 × 10^9^), of hemoglobin (Hb) was 107000 mg/L (112–1620000), of Glasgow coma scale (GCS) was 7 (6–9), of PCV was 38.1% (30.1–48.4), of alanine transaminase (ALT) was 214 U/L (36.3–123000), of aspartate transaminase (AST) was 185 U/L (44–199000), of albumin was 3.05 g/dL (2.3–4.2), of creatine was 1.2 mg/dL (0.6–4.3), of glucose was 103.8 mg/dL (69–138), of urea was 46 mg/dL (21.9–102) (S6 Table).

The optic nerves were unable to be examined (3 cases), normal (3 cases), B/L ptosis (1 case), optic neuritis (1 case), B/L involvement (1 case), moderate B/L optic atrophy (1 case) and ND (19 cases). The fundus examination was normal in 9 cases, showed pallor of optic discs in 1 case, B/L papilledema which was more severe in the right eye in another case and ND in 18 cases. Pupils have normal size and reaction to light in 5 cases, sluggish reaction to light in 1 case, mid-dilated and equal in size with sluggish reaction to light in 1 case, mid-dilated, symmetrical with sluggish reaction to light in 1 case, dilated in 1 case, B/L mid-dilated, symmetrical and sluggishly reacting to light in another case and ND in 19 cases. The visual acuity was deteriorated in left eye then in right eye then in both eyes gradually deteriorated in 1 case, another case had a severe visual impairment in right eye (only light perception) and a slight visual disturbance in left eye (VA = 20/25), 1 case with a severe right visual impairment and ND in 26 cases (S7 Table).

### Treatment and outcomes

The follow-up period ranged from 28 days to 5 years (S2 Table). Most specific treatments used for ADEM were oral or intravenous (IV) corticosteroids including methylprednisolone (11 cases), prednisolone (7 cases) and dexamethasone (5 cases) or human immunoglobulin (1 case). Other treatments used were anticonvulsant medications such as phenytoin (2 cases) and phenobarbitone (1 case), oral or intravenous antipyretics and anticonvulsant such as dipyrone, paracetamol, pulse therapy, dopamine, noradrenaline, and lorazepam (1 case for each treatment). The outcomes in these cases were either death (3 cases), partial recovery (7 cases), complete recovery (16 cases) or ND (3 cases). The cases with partial recovery were either; had mild B/L visual disturbance, dysuria, and dyschezia remained [49], was able to walk with a minimal support [50], wanted to carry further treatment in the hospital [51], a slight residual cerebellar ataxia [52], the frontal symptoms persisted [53], mild ataxia and dysarthria [54]. The three cases died due to; myalgia, jaundice, conjunctival hemorrhage, hematuria, oliguria, shortness of breath, became stuporous, acute respiratory distress syndrome (ARDS), acute kidney injury and metabolic acidosis [55], intracranial tension [56] or B/L hemorrhagic demyelination [57] (Table 2).

**Table 2:** The main manifestations, treatments used for ADEM and outcomes of the included cases.

Author, year	Main manifestations	Treatment of ADEM	Outcome
**Pal, 2016 [44]**	Altered sensorium and quadriparesis	IV methylprednisolone 1 g once daily for 5 days	Complete recovery: he regained his power in ULs, but both LL had 4/5 power on discharge. At first follow-up, after 3 weeks of discharge, he had no residual paraparesis.
**Moura, 2004 [43]**	ND	ND	Complete recovery
**Abdulrazak, 2015 [75]**	Fever and urine problems	Phenobarbitone and phenytoin	Complete recovery: fever resolved, sensorium normalized
**Sundaram, 2010 [55]**	Fever and urine problems	Mechanical ventilation and managed for ADRS, peritoneal dialysis for acidosis and kidney injury	Died
**Singh, 2015 [76]**	Comatose and convulsion	Midazolam and loading dose of phenytoin, IV methylprednisolone then oral prednisolone	Complete recovery
**Gera, 2010, [77]**	Fever and convulsion	High dose of corticosteroids	Complete recovery: at 8 months follow up, he was asymptomatic with no neurological deficits
**Gupta, 2013 [61]**	Comatose	1 g IV methylprednisolone for 5 days	Complete recovery
**Yamamoto,** **2002 [49]**	B/L sensory disturbance and paraplegia visual disturbance	1 g methylprednisolone for 3 days, then 3 times per week	Partial recovery: mild B/L visual disturbance, dysuria, and dyschezia remained
**Kumar, 2014 [78]**	Fever and severe hypoxemia and septicemia	ND	Partial recovery; he was discharged after 33 days of hospitalization with improving right hemiparesis and he was able to walk with minimal support.
**Brito, 2007 [79]**	Gait problems	Dipyrone, pulse therapy with methylprednisolone	ND
**Bhat, 2010 [51]**	Fever and intermittent decerebrate posture	30 mg/kg/d methylprednisolone	Partial recovery; his attendants wanted to carry further treatment in a hospital at their native place, so he was transferred there.
**Chakrabarti, 2015 [52]**	Fever and gastric tenderness	IV lorazepam, phenytoin, IV ceftriaxone, dexamethasone	Partial recovery; he gradually improved on treatment and was discharged in stable condition with slight residual cerebellar ataxia
**Cunha-Matta, 2004 [53]**	Fever and slurred speech	Paracetamol	Partial recovery; the frontal symptoms persisted at four months follow-up
**Chowdhury, 2011 [54]**	Severe low backache and headache	Anticonvulsant, dexamethasone, IV methylprednisolone, dexamethasone, oral prednisolone, diazepam & phenobarbitone	Partial recovery; she gradually improved and regained her consciousness on the 8th day after admission. On discharge, the patient was conscious, oriented with a residual headache, ataxia, and dysarthria. Platelet count and laboratory parameters became normal. At follow-up after 2 months, her headache had subsided but mild ataxia and dysarthria were still present.
**Gupta, 2015 [68]**	Sensations diminished below D10 segment	IV dexamethasone & steroids	Complete recovery; within 48 hours, he started showing improvement. He was able to walk with some support by five days. He was discharged after six days of admission, and then he came for follow-up, for a period of one week. He showed progressive improvement and could pass urine without a catheter. He was then advised a tapering dose of steroids
	Fever, urine problems	IV dexamethasone	Complete recovery; gradually, he started improving and at the time of discharge five days after admission, the patient was able to walk and pass urine without a catheter. B/L ptosis had also improved.
**Dewan, 2016 [56]**	Fever, generalized maculopapular rash	ND	Died
**Gala, 2012 [80]**	Fever, aggressive behavior and hallucinations	Phenytoin, phenobarbitone, IV valprin, IV methylprednisolone, oral prednisolone, dopamine, nor-adrenaline infusion	Complete recovery; she was neurologically normal at discharge
**Verma, 2011[81]**	Fever and spasticity in LL	ND	Complete recovery
**de Sousa, 2006 [42]**	Fever, B/L pyramidal syndrome, and paraparesis	IV methylprednisolone & oral prednisone	Complete recovery; remission of paraplegia and visual deficit, normal visual acuity in left eye, mild visual dysfunction in right eye (with pale papilla) which recovered later on
**Koshy, 2012 [57]**	Hemiparesis	ND	Died
	Facial nerve palsy and quadriparesis	ND	Complete recovery
**Karoli, 2016 [82]**	Fever and convulsion	IV methyl prednisolone & oral prednisolone	Complete recovery; rapid clinical improvement was noticed. The repeat MRI after two weeks also showed almost complete resolution of patchy demyelinating lesions
**Puccioni-Sohler, 2009 [62]**	Fever and urine problems	Methylprednisolone & human immunoglobulin	Complete recovery; visual function was recovered, and he could walk in 10 months
**Pan, 2016 [83]**	Fever and urine problems	IV steroids with bedside physiotherapy & supportive measures	Complete recovery; a dramatic improvement over the next few weeks. CSF was repeated after two weeks which revealed glucose 50 mg/dl, protein 58 mg/dl, cell count 3/cm (all lymphocytes). He was able to walk independently at the end of four weeks
**Fragoso, 2016 [84]**	Urine problems	IV corticosteroids	Complete recovery after 5 years five years later, his general health status was good, although he continued to present discrete right hemiparesis (upper and lower right limbs), hyperreflexia, and proprioceptive sensory deficits in both feet.
**Ferreira, 2005 [45]**	Unclear*	ND	ND
**Wasay, 2008 [28]**	Unclear*	ND	ND
**Our present case**	Fever and urine problems	Methylprednisolone, oral prednisone	Partial recovery; mild weakness in the proximal part of her legs

Abbreviations; ND = Not described, ULs = Upper limbs, IV = intravenous, B/L = bilateral.

*Unclear means that the information in this paper, which contained more than one case, were not specified for ADEM case.

Our results showed that the body temperature levels in the complete recovery group were significantly lower than those of the partial recovery (p-value = 0.026) and bad outcomes groups (p = 0.03). While there was a significant difference between cases having complete recovery or bad outcomes on the onset day of neurological manifestations which was found started earlier in cases having partial recovery (p = 0.03) and bad outcomes (p = 0.006) as compared to patients with complete recovery. Other factors including gender, steroid treatment, and its administration route, age, hemoglobin, platelet, WBC, GCS, PCV, ALT, AST, pulse, systolic blood pressure, diastolic blood pressure, urea, glucose, creatinine or albumin were not associated with the ADEM outcomes (Table 3).

**Table 3:** The association of patients’ characteristics and manifestations with ADEM cases’ outcomes.

Factor	Comparison groups				
	Complete recovery (n = 16)*	Partial recovery (n = 7)*	P-value^a^	Bad outcomes (Death or partial recovery) (n = 10)*	P-value^b^
Gender (male), (n) (%)**	11 (68.8)	5 (71.4)	1	8 (80)	0.67
Steroids treatment, (n) (%)**	11 (68.8)	5 (71.4)	1	5 (50)	0.43
Administration route (IV), (n) (%)**	6 (37.5)	3 (42.8)	1	3 (30)	1
Age (years)	22.5 (9 days– 65 years)	15 (10–58)	0.76	17 (10–58)	0.97
Hb (mg/L)	10 × 10^4^ (112–113 × 10^3^)	63 (119–12 × 10^4^)	0.56	117500 (119–162 × 10^3^)	0.18
Onset day of neurological manifestations	**11 (5–19)**	**6 (5–8)**	**0.03**	**5.5 (3–8)**	**0.006**
Platelet (L)	655 × 10^8^ (18 × 10^9^–19 × 10^12^)	60 × 10^9^ (20 × 10^9^–328 × 10^9^)	0.92	45 × 10^9^ (16 × 10^9^–328 × 10^9^)	0.43
WBC (L)	51 × 10^8^ (9500–31 × 10^9^)	4 × 10^9^ (0.011–75 × 10^8^)	0.49	41 × 10^9^ (0.011–146 × 10^8^)	0.95
GCS	7.5 (7–8)	7.5 (6–9)	1	7 (6–9)	0.9
PCV (%)	31.5 (37.9–48.4)	38.2 (37.9–48.4)	0.23	42.1 (37.9–48.4)	0.21
ALT (U/L)	211.5 (36.3–1457)	313 (43–123000)	0.87	214 (38–123000)	0.80
AST (U/L)	156.5 (77.9–832)	9232.5 (44–199000)	0.85	465 (44–199000)	1
Temperature (°F)	**100 (99–102)**	**102.2 (100.4–105)**	**0.026**	**102.2 (100.4–105)**	**0.03**
Pulse (per minute)	105 (100–110)	110 (100–140)	0.67	110 (92–140)	0.95
Systolic blood pressure (mmHg)	100 (90–110)	98 (90–130)	0.97	100 (90–130)	0.99
Diastolic blood pressure (mmHg)	65 (60–70)	70 (59–79)	0.66	60 (60–80)	1
Urea (mg/dl)	24 (21.9–108)	69 (26–84)	0.38	69 (26–84)	0.29
Glucose (mg/dl)	102.4 (69–138)	100.8	1	105.4 (100.8–110)	0.8
Creatinine (mg/dl)	1.15 (1–1.9)	1.2 (0.9–1.6)	0.94	1.2 (0.6–4.3)	0.95
Albumin (g/dl)	2.75 (2.3–3.2)	3.55 (2.9–4.2)	1	3.55 (2.9–4.2)	1

Abbreviations; Vs. = Versus, IV = Intravenous, Hb = Hemoglobin, GCS = Glasgow coma scale, PCV = packed cell volume, AST = Aspartate transaminase, ALT = Alanine transaminase.

^a^P-value for comparison between complete recovery versus partial recovery.

^b^P-value for comparison between complete recovery versus bad outcomes.

*The numbers are median and range except for gender, steroids treatment and administration route which are the frequency and percent.

**The test used for all variables was Mann-Whitney U test except for gender, steroids treatment and administration route (Fixer’s exact test).

### Classification tree models

**Fig 5:**
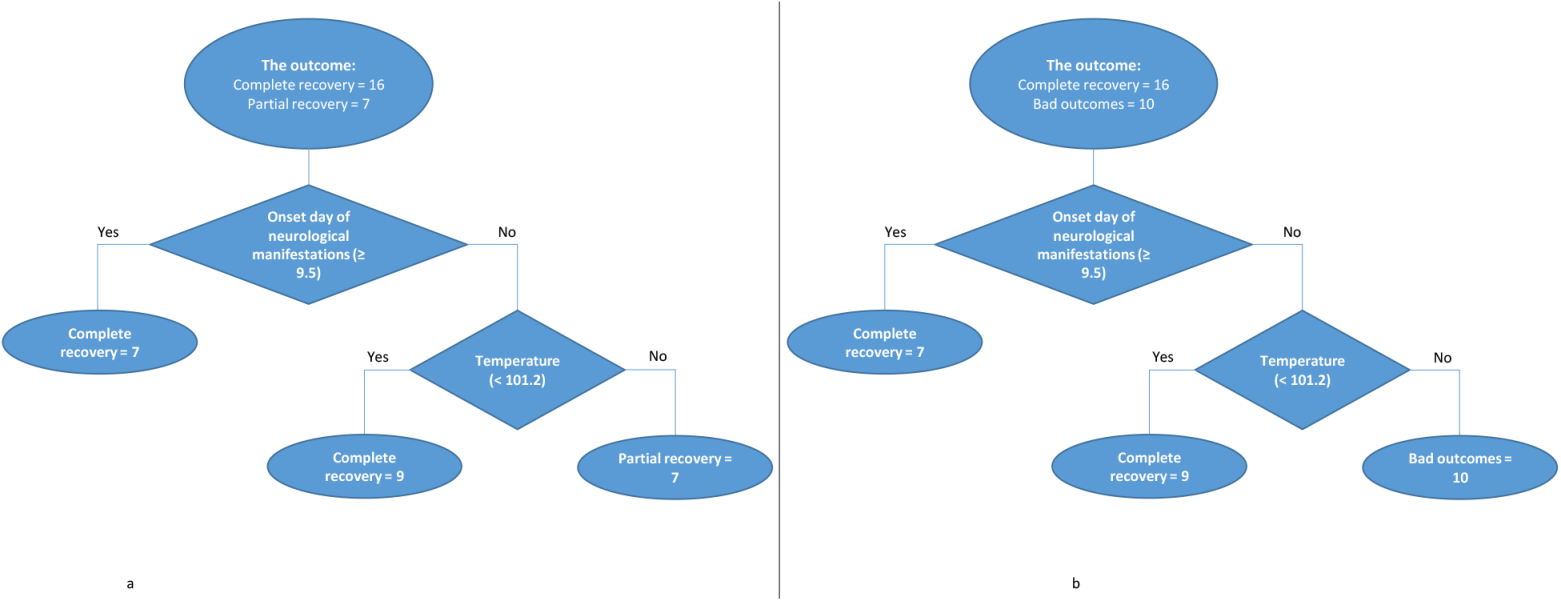
**Classification tree models indicating the significant predictors for:** partial recovery (A) bad outcomes (B) of ADEM compared with complete recovery.

We then selected all of the aforementioned 20 variables to build classification tree models for bad outcomes (partial recovery or died) versus complete recovery and for partial recovery versus complete recovery. Interestingly, that both classification trees including the onset day of neurological manifestations (the cut-off point at 9.5 days), and temperature (the cut-off point at 101.2°F) are the best models (Fig 5). Both trees enhance the results from uni-variable analysis indicating that the earlier onset day of the neurological manifestations (< 9.5 days) and higher fever when presenting ADEM (≥ 101.2°F) were associated with the bad outcomes and partial recovery. The performance of tree that classified bad outcomes versus complete recovery is at the accuracy of 84.6% [65.1–95.6%] with the sensitivity of 80%, specificity of 87.5%, PPV of 80% and NPV of 87.5%. Whereas, the performance of tree that classified complete recovery versus partial recovery is at the accuracy of 82.6% [61.2–95.1%] with the sensitivity of 87.5%, specificity of 71.4%, PPV of 87.5% and NPV of 71.4% (Fig 5).

## Discussion

Our meta-analysis revealed that the prevalence of patients with neurological disorders within dengue patients is not rare (2.6%). Though ADEM was reportedly stated as a rare condition [57–59], the incidence could be higher because of the high global burden of dengue infection. In a previous study, it revealed that dengue was present in 4–47% of patients with encephalitis in the endemic regions [60]. It is well-known that encephalopathy is the most common neurological disorder accompanying DENV infection [47, 48]. Thus, this should push health cares to estimate the total global cases of ADEM from the annual incidence of dengue because the number of post-dengue ADEM is underestimated probably due to the neglect of the clinicians and patients, hence our current proposal is to screen all patients with neurological manifestations against dengue and most other flaviviruses such as Zika to investigate the number of ADEM cases in a multi-center study in Vietnam and Philippines. Moreover, we suggest adding such neurological complications in the dengue WHO guidelines, so they get no neglect.

The onset of ADEM ranged from day 3 to day 19 from dengue infection. The clinicians should be aware that patients can present with early or late onset of ADEM symptoms and patients may not always mention a recent history of fever. Moreover, there was a significant difference between cases having complete recovery or bad outcomes only in two factors which were the onset day of the neurological manifestations being earlier and the temperature being higher in cases having bad outcomes or partial recovery through our uni-variable analysis. This finding is supported by classification tree models including the onset day of the neurological manifestations and temperature. Both trees indicate that the earlier onset day of the neurological manifestations (< 9.5 days) and higher fever when presenting ADEM (≥ 101.2°F) were associated with the bad outcomes. These findings require an attention from physicians regarding the temperature of the dengue cases to be managed well once elevated.

The most frequent manifestations related to dengue infection arranged from the most frequent to the least frequent were; fever, thrombocytopenia, and vomiting, headache, erythema/rash, myalgia, arthralgia, chills, leukocytopenia, and restlessness then retro-orbital pain, rigors, and lethargy. Noteworthy, vomiting, rash, and leukocytopenia are classified as dengue without warning signs in the WHO 2009 guidelines while thrombocytopenia, restlessness, and lethargy are classified as dengue warning signs [1]. While the main manifestations related to ADEM arranged from the most frequent to the least frequent were; altered sensorium/consciousness, seizures and urination problems, vision problems, slurred speech, walk problems, then ataxia. Similarly, altered consciousness is classified within the severe dengue signs, in the severe CNS involvement, in the WHO 2009 guidelines. However, other ADEM manifestations are not mentioned in it [1], maybe because ADEM sometimes appears late after dengue. Hence, we suggest adding them to the guidelines of severe organs involvement stage.

The results from MRI of the brain and spinal cord showed that most of the cases have abnormalities such as T2 lesions. In contrast to our results which showed no abnormalities. The attention for the MRI of either the brain or spinal cord findings should be paid more due to its unlimited importance in diagnosis and treatment of ADEM cases. Most of the outcomes in these cases were relatively good because most of them showed either partial recovery or complete recovery. There was no significant difference between cases with bad outcomes or complete recovery in the treatment used. Unlike a previous literature review which suggested that steroids are promising in the treatment of ADEM during its active phase [61].

Till now, there is no study described the mechanism of post-dengue ADEM. The neurological complications of dengue infection have been considered to be due to systemic complications of dengue and not related to its neurotropic nature [48, 61–65]. After the demonstration of neural tropism of dengue virus, the neurological manifestations of dengue infection are categorized as (1) related to direct neurotropic effects of the virus (myelitis, meningitis, myositis, rhabdomyolysis, and encephalitis), (2) related to systemic or metabolic complications of dengue (encephalopathy, stroke) and (3) post-infectious immune-mediated complications (GBS, transverse myelitis, ADEM). ADEM usually occurs following a viral infection but may appear spontaneously, after bacterial, parasitic infection or vaccination. Most cases follow a nonspecific upper respiratory tract infection. Although it occurs in all ages, most reported cases are in infants and adolescents [66]. The post-infectious ADEM usually begins late in the course of viral infections including measles, chickenpox, mumps, rubella, influenza, EBV and nonspecific respiratory infections. The pathophysiology involves a transient auto-immune response directed at myelin or other self-antigens, possibly by a non-specific activation of auto-reactive T-cell clones or by molecular mimicry [63, 67, 68]. As with other viruses, the pathogenesis underlying dengue-associated ADEM may result from an immune system mediated-process [25].

A limitation of this study was the small number of included studies (a total of 29 ADEM cases) and reported cases, with some missing values, included in the uni-variable analysis, meta-analysis, and the classification tree models. Moreover, our results should be interpreted with caution because most cases depended on IgM ELISA which has a probable diagnosis [1] but with a high specificity [1, 69–74]. In conclusion, our analysis of the case report and other included cases revealed that the onset day of neurological manifestations and temperature in the ADEM patients were associated with the disease outcome and can predict it. Moreover, we found that the most frequent dengue manifestations were fever, thrombocytopenia, vomiting, and headache while the most frequent ADEM manifestations were altered sensorium/consciousness, seizures urination problems, and vision problems. The serious manifestations after dengue infection continue to be reported. These manifestations should be considered in the diagnosis and management of patients with dengue infection. The prevalence of ADEM among dengue and other dengue-related neurological disorders is not too rare. Since the incidence of ADEM is not known well, future larger studies are necessary to accurately investigate ADEM.

## Supporting information

S1 Fig Axial non-contrast T1-weighted MRI. (DOCX)

S2 Fig Axial non-contrast T2-weighted MRI. (DOCX)

S3 Fig Axial contrast-enhanced T1-weighted MRI. (DOCX)

S4 Fig Nerve conduction examination. (DOCX)

S5 Fig Electromyography (EMG). (DOCX)

S1 Table PRISMA 2009 checklist. (DOCX)

S2 Table The frequency of each manifestation. (DOCX)

S3 Table Renal and liver function tests, urinalysis, tourniquet test, abdominal pressure, cardiovascular system, abdominal findings, temperature, respiratory rate (RR), pulse, chest X-rays, follow-up period and onset day of ADEM/discharge. (DOCX)

S4 Table Cerebrospinal fluid (CSF), magnetic resonance imaging (MRI) of the brain and spinal cord results. (DOCX)

S5 Table Power grade, cranial nerves, motor system examination. (DOCX)

S6 Table Blood examination results. (DOCX)

S7 Table Visual examination. (DOCX)
