# Supplementary figures and images for: Post-dengue acute disseminated encephalomyelitis: A case report and meta-analysis
Source: PLoS Negl Trop Dis. 2017 Jun 30;11(6):e0005715. doi: 10.1371/journal.pntd.0005715 (PMC5509372; doi:10.1371/journal.pntd.0005715)

**S1 Fig. Axial non-contrast T1-weighted MRI.**

**
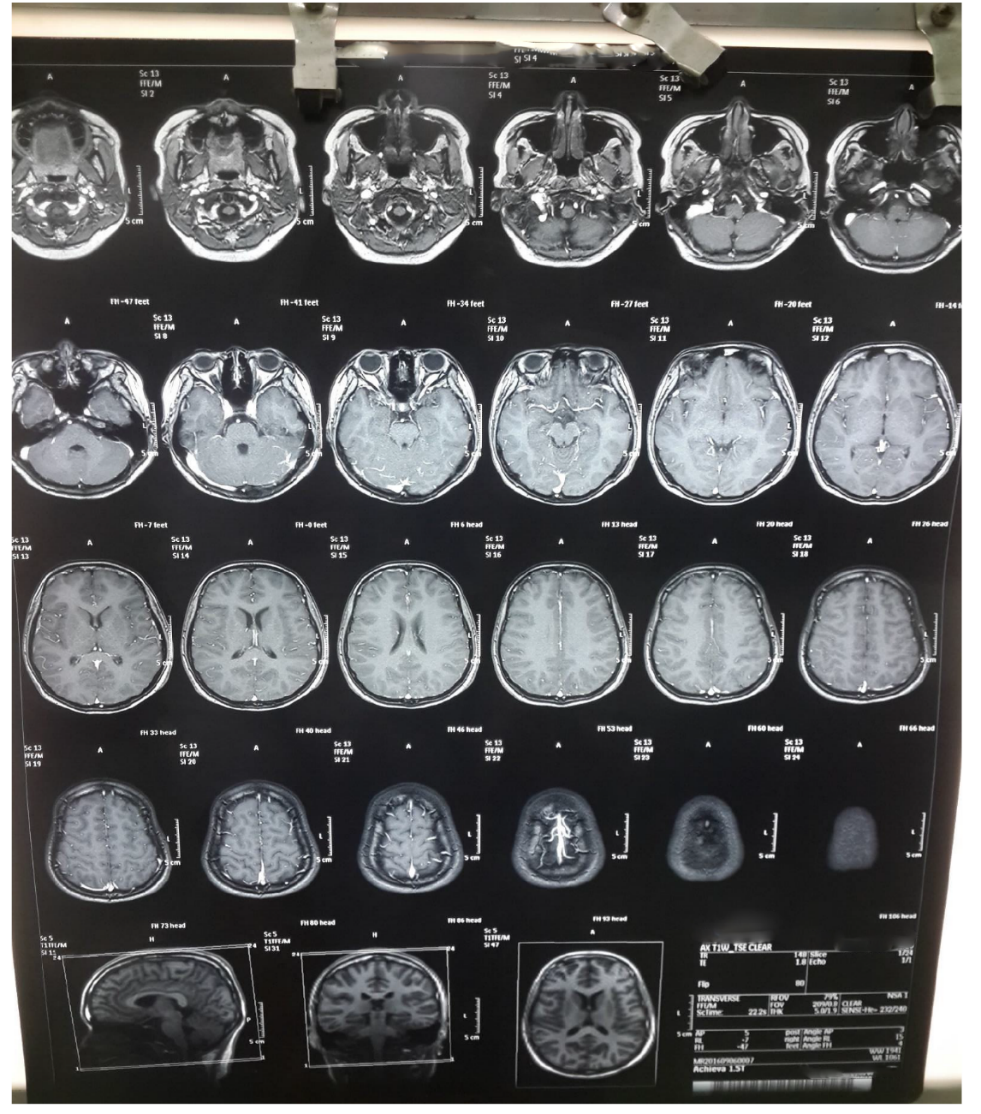
**

Supplement: S1 Fig — (DOCX) [file pntd.0005715.s001.docx]

**S2 Fig. Axial non-contrast T2-weighted MRI.**

*
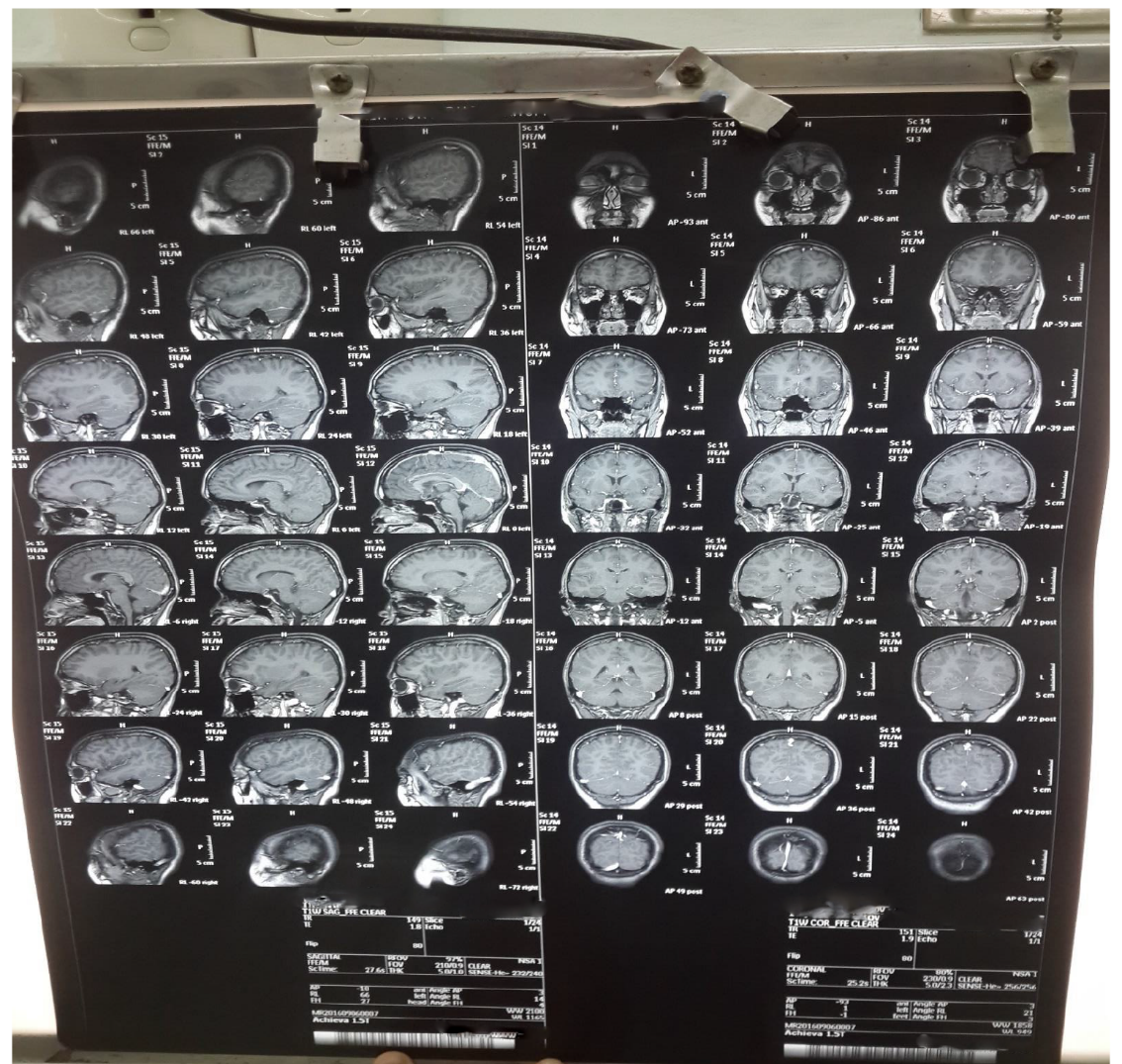
*

Supplement: S2 Fig — (DOCX) [file pntd.0005715.s002.docx]

**S3 Fig. Axial contrast-enhanced T1-weighted MRI.**

*
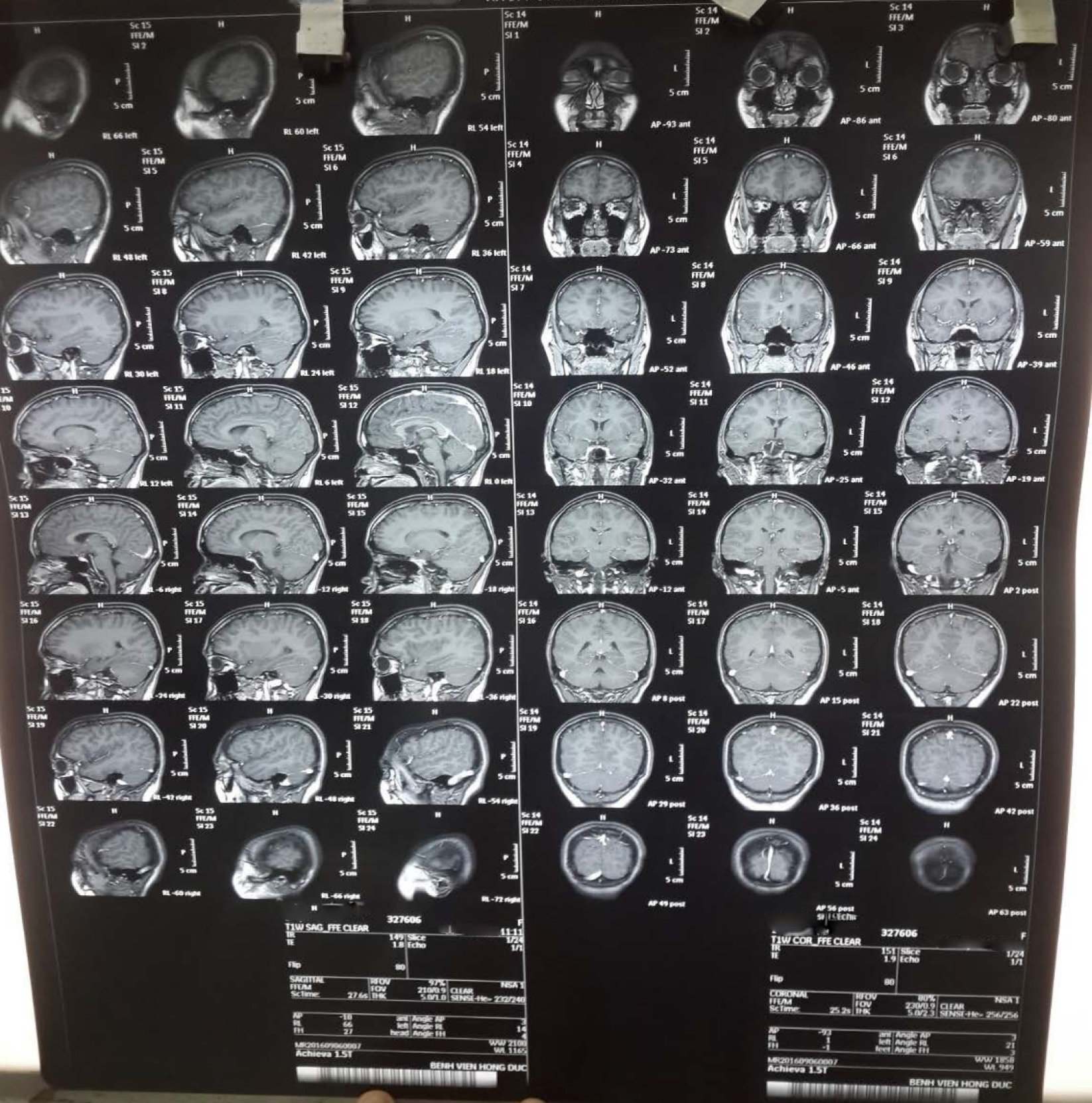
*

Supplement: S3 Fig — (DOCX) [file pntd.0005715.s003.docx]

**S4 Fig. Nerve conduction examination.**

***
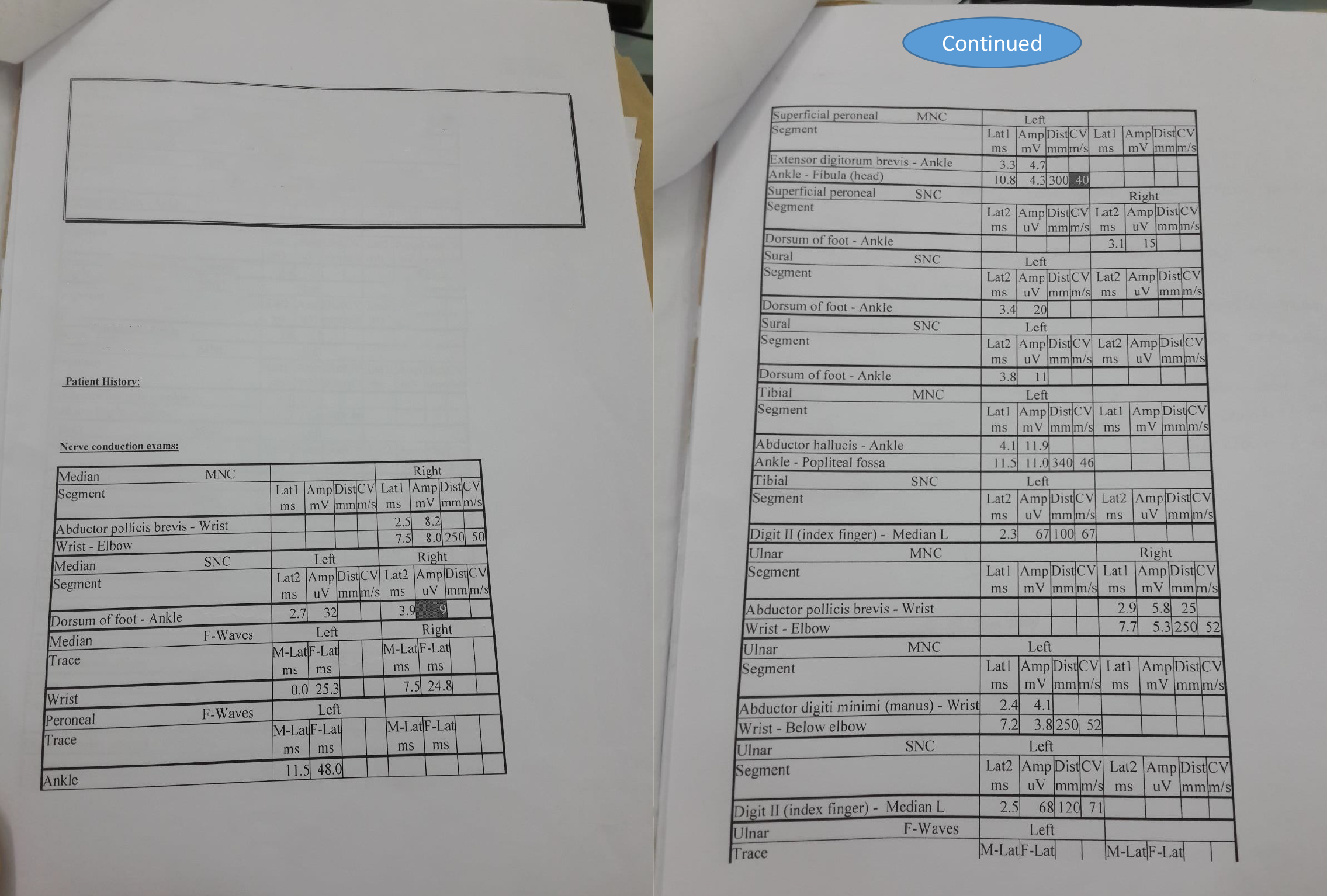
***

Supplement: S4 Fig — (DOCX) [file pntd.0005715.s004.docx]

**S5 Fig. Electromyography (EMG).**


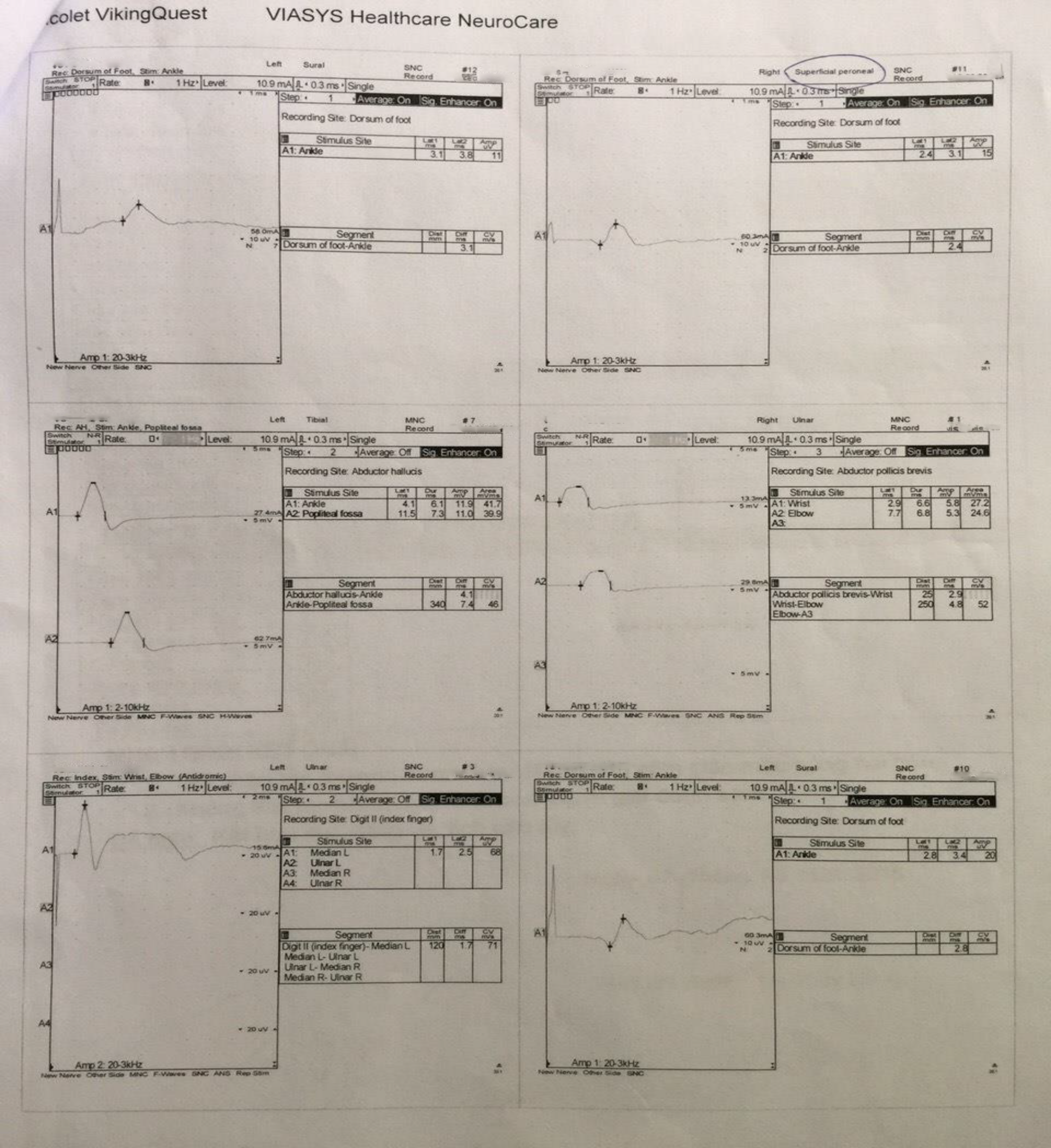

Supplement: S5 Fig — (DOCX) [file pntd.0005715.s005.docx]
