# Supplementary material for: Post-dengue acute disseminated encephalomyelitis: A case report and meta-analysis
Source: PLoS Negl Trop Dis. 2017 Jun 30;11(6):e0005715. doi: 10.1371/journal.pntd.0005715 (PMC5509372; doi:10.1371/journal.pntd.0005715)
Supplement: S2 Table — (DOCX) [file pntd.0005715.s007.docx]

**S2 Table. The frequency of each manifestation.**

| Manifestations | Frequency in 26 cases (including our case)* | Prevalence (%) |
| --- | --- | --- |
| Dengue-related Manifestations | | |
| Fever | 22 | 85 |
| Thrombocytopenia | 13 | 50 |
| Vomiting |  |  |
| Headache | 11 | 42 |
| Erythema /rash | 9 | 35 |
| Myalgia | 8 | 31 |
| Arthralgia | 6 | 23 |
| Chills | 5 | 19 |
| Leukocytopenia | 4 | 15 |
| Restless | 4 |  |
| Lethargy | 3 | 12 |
| Rigors |  |  |
| Retro-orbital pain |  |  |
| ADEM-related Manifestations | | |
| Altered sensorium/consciousness | 15 | 58 |
| Seizures | 9 | 35 |
| Urination problems |  |  |
| Vision problems | 8 | 31 |
| Slurred speech | 6 | 23 |
| Walk problems | 4 | 15 |
| Ataxia | 3 | 12 |

**The frequency was calculated in 25 cases + our case only (26 cases) not in 28 cases + our case (29 cases) due to lack of information in three cases.*
