# Supplementary material for: Post-dengue acute disseminated encephalomyelitis: A case report and meta-analysis
Source: PLoS Negl Trop Dis. 2017 Jun 30;11(6):e0005715. doi: 10.1371/journal.pntd.0005715 (PMC5509372; doi:10.1371/journal.pntd.0005715)
Supplement: S3 Table — (DOCX) [file pntd.0005715.s008.docx]

**S3 Table. Renal and liver function tests, urinalysis, temperature, respiratory rate (RR), pulse, day of post-dengue ADEM onset/discharge and follow-up time.**

| Author, year | Urinalysis | Renal Function test | Liver Function test | RR/min | TT | ABP (mm/Hg) | Pulse (beats/min) | Temp. (^o^F) | Abdominal findings | CVS | Day of post-dengue ADEM onset/discharge^*^ | Follow-up time* | Chest X-ray |
| --- | --- | --- | --- | --- | --- | --- | --- | --- | --- | --- | --- | --- | --- |
| Pal, 2016 [1] | ND | ND | N | ND | ND | 100/60 | 110 | 101 | ND | ND | 7/4 months | 4 months | ND |
| Moura, 2004 [2] | ND | ND | ND | ND | ND | ND | ND | ND | ND | ND | ND/ND | ND | ND |
| Abdulrazak, 2015 [3] | ND | ND | ND | ND | ND | ND | ND | ND | ND | ND | ND/12 | 85 | ND |
| Sundaram, 2010 [4] | ND | Acute kidney injury and metabolic acidosis | ND | Stable | ND | Stable | Stable | Stable | Mild hepatomegaly | ND | 5/NA | ND | B/L fluffy shadows and ARDS |
| Singh, 2015 [5] | ND | ND | ND | ND | ND | ND | ND | ND | ND | ND | 14/ND | ND | ND |
| Gera, 2010, [6] | ND | ND | ND | ND | ND | ND | ND | ND | ND | ND | 6/8 months | 8 months | ND |
| Gupta, 2013 [7] | ND | Urea: 46 mg/dl, Creatinine 1.1 mg/dl | ALT= 96 U/l, AST= 128 U/l | ND | ND | ND | ND | 99.6 °F | ND | ND | 6/ND | ND | ND |
| Yamamoto, 2002 [8] | ND | N | Abnormal then N (ALT= 123000 U/l, AST= 199000 U/l) | ND | ND | ND | ND | Over 39 °C | ND | ND | 7/69 | ND | ND |
| Kumar, 2014 [9] | ND | ND | ALT= 313 U/l, AST= 465 U/l | 28 | ND | 130/80 | 110 | 101.4 °F | ND | ND | 5/33 | 33 | ND |
| Brito, 2007 [10] | ND | ND | N (ALT= 19000 U/l, AST= 18000 U/l) | ND | ND | ND | ND | ND | ND | ND | 5/ND | ND | ND |
| Bhat, 2010 [11] | ND | ND | ND | 30 | ND | 100/80 | 140 | 38 C | ND | ND | 6/ND | ND | ND |
| Chakrabarti, 2015 [12] | N | N | N | ND | + | 96/60 | 110 | 103 °F | N | ND | 6/ND | ND | N |
| Cunha-matta, 2004 [13] | ND | ND | ALT= 43 U/l, AST= 44 U/l | ND | ND | ND | ND | 39-40 C | ND | ND | 7/120 | ND | ND |
| Chowdhury, 2011 [14] | N | ND | ALT= 70 U/l, AST= ND | ND | + | 90/60 | 100 | 105 °F | ND | ND | 5/ND | 2 months | N |
| Gupta, 2015 [15] | ND | ND | ALT= 79.3 U/L, AST= 119.7 U/L | ND | ND | 110/70 | ND | 99 °F | Distended UB, splenomegaly 2 CMs and hepatomegaly 3 CMs | N | 7/13 | ND | N |
|  | N | ND | ALT= 36.3 U/L, AST= 77.9 U/L | ND | ND | 100/70 | ND | 100 °F | Markedly distended and palpable UB and spleen tip palpable | N | ND/6 | ND | N |
| Dewan, 2016 [16] | ND | ND | ALT= 38 U/L, AST= 95 U/L | ND | ND | 100/60 | 92 | ND | ND | ND | 3/ND | ND | ND |
| Gala, 2012 [17] | ND | N | N | ND | ND | ND | ND | ND | ND | ND | 12/ND | ND | ARDS |
| Verma, 2011[18] | ND | ND | ND | ND | ND | Stable | Stable | ND | ND | ND | 5/ND | ND | ND |
| de Sousa, 2006 [19] | ND | ND | ND | ND | ND | ND | ND | ND | ND | ND | 15/ND | ND | ND |
| Koshy, 2012 [20] | ND | ND | ALT= 115 U/L, AST= 139 U/L | ND | ND | ND | ND | ND | ND | ND | ND/ND | ND | ND |
|  | ND | ND | ALT= 327 U/L, AST= 185 U/L | ND | ND | ND | ND | ND | ND | ND | ND/ND | ND | ND |
| Karoli, 2016 [21] | ND | ND | ND | ND | ND | ND | ND | ND | Pain | ND | 19/ND | ND | ND |
| Puccioni-Sohler, 2009 [22] | ND | ND | ND | ND | ND | ND | ND | ND | ND | ND | 12/10 months | Unclear | ND |
| Pan, 2016 [23] | ND | ND | ND | ND | ND | ND | ND | ND | ND | ND | 8/28 | 28 | ND |
| Fragoso, 2016 [24] | ND | ND | ND | ND | ND | ND | ND | ND | ND | ND | 14/ND | 5 years | ND |
| Ferreira, 2005 [25] | ND | ND | ND | ND | ND | ND | ND | ND | ND | ND | ND/ND | ND | ND |
| Wasay, 2008 [26] | ND | ND | ND | ND | ND | ND | ND | ND | ND | ND | ND/ND | ND | ND |
| Our present case | ND | N | N | ND | ND | ND | ND | ND | ND | ND | 8/30 | 30 | ND |

Abbreviations; N = Normal, + = Positive, - = Negative, B/L = Bilateral, ND = Not described, ARDS = acute respiratory distress syndrome, ALT = Alanine Aminotransferase, AST = Aspartate Aminotransferase, Temp = temperature, RR =respiratory rate, TT = tourniquet test, CVS =cardiovascular system, ABP = arterial blood pressure, Min = minute, UB = Urinary Bladder.

*Age is in years, while the day of post-dengue ADEM onset/discharge and follow-up are in days, otherwise stated.

**References**

1. Pal S, Sen K, Biswas NM, Ghosal A, Jaman SR, Kumar KY. Clinico-radiological profile and outcome of dengue patients with central nervous system manifestations: A case series in an Eastern India tertiary care hospital. J Neurosci Rural Pract. 2016;7(1):114.

2. Moura P, Cordeiro MT, Brito C, Lima Filho JLd, Arraes LC. Neurological aspects of the dengue infection in Brazilian patients during the DENV3 epidemicas. An Fac Med Univ Fed Pernamb. 2004;49(2):115-8.

3. Razak AN, N. K. Acute Demyelinating Encephalomyelitis in a Neonate Secondary to Dengue Infection. Indian Pediatr. 2015;52(6):534. Epub 2015/06/30. PubMed PMID: 26121739.

4. Sundaram CU, S. G.; Dakshinamurthy, K. V.; Borgahain, R. Acute disseminated encephalomyelitis following dengue hemorrhagic fever. Neurol India. 2010;58(4):599-601. Epub 2010/08/27. doi: 10.4103/0028-3886.68666. PubMed PMID: 20739802.

5. Singh GS, Gursharan; Arora, Sunita. Acute Disseminated Encephalomyelitis Following Dengue Infection. 2015.

6. Gera CG, U. Acute disseminating encephalomyelitis with hemorrhage following dengue. Neurol India. 2010;58(4):595-6. Epub 2010/08/27. doi: 10.4103/0028-3886.68661. PubMed PMID: 20739799.

7. Gupta M, Nayak R, Khwaja GA, Chowdhury D. Acute disseminated encephalomyelitis associated with dengue infection: a case report with literature review. J Neurol Sci. 2013;335(1):216-8.

8. Yamamoto Y, Takasaki T, Yamada K-i, Kimura M, Washizaki K, Yoshikawa K, et al. Acute disseminated encephalomyelitis following dengue fever. J Infect Chemother. 2002;8(2):175-7.

9. Sanjeev Kumar BN, S; Jayantee, K; Misra, UK. Acute Disseminated Encephalomyelitis following Dengue Virus Infection. J Neuroinfect Dis. 2014;5(139):2.

10. Brito CA, Sobreira S, Cordeiro MT, Lucena-Silva N. Acute disseminated encephalomyelitis in classic dengue. Rev Soc Bras Med Trop. 2007;40(2):236-8.

11. Bhat D. Acute disseminated encephalomyelitis: A rare central nervous system manifestation of dengue hemorrhagic fever. Journal of Pediatric Infectious Diseases. 2010;5(4):415-7.

12. Chakrabarti S. A case of acute disseminated encephalomyelitis following dengue infection. CHRISMED Journal of Health and Research. 2015;2(2):169.

13. Palma da Cunha-Matta ASM, S. A.; Cardoso de Almeida, A.; Aquilera de Freitas, V.; Carod Artal, F. J. Complicaciones neurológicas de la infección por el virus del dengue. Rev Neurol. 2004;39(3):233-7.

14. Chowdhury R, Siddiqui M, Mahbub M, Hasan O, Talukder A, Nabi S, et al. Dengue fever as a cause of acute disseminated encephalomyelitis (ADEM). J Med. 2011;12(2):185-7.

15. Gupta R, Gupta P, Sharma R. Dengue fever presenting as acute disseminated encephalomyelitis (ADEM). Journal, Indian Academy of Clinical Medicine. 2015;16(2):159.

16. Dewan R, Anuradha S, Sethi P, Ish P. Dengue presenting as hemorrhagic acute disseminated encephalomyelitis. MAMC Journal of Medical Sciences. 2016;2(1):54.

17. Gala HC, Avasthi BS, Lokeshwar MR. Dengue shock syndrome with two atypical complications. The Indian Journal of Pediatrics. 2012;79(3):386-8.

18. Verma R, Sharma P, Garg RK, Atam V, Singh MK, Mehrotra HS. Neurological complications of dengue fever: Experience from a tertiary center of north India. Annals of Indian Academy of Neurology. 2011;14(4):272.

19. de Sousa AM, Puccioni-Sohler M, Borges AD, Adorno LF, Alvarenga MP, Alvarenga RMP. Post-dengue neuromyelitis optica: case report of a Japanese-descendent Brazilian child. J Infect Chemother. 2006;12(6):396-8.

20. Koshy JM, Joseph DM, John M, Mani A, Malhotra N, Abraham GM, et al. Spectrum of neurological manifestations in dengue virus infection in Northwest India. Trop Doct. 2012;42(4):191-4.

21. Karoli R, Siddiqi Z, Fatima J, Maini S. Was it a case of acute disseminated encephalomyelitis? A rare association following dengue fever. J Neurosci Rural Pract. 2013;4(3):318.

22. Puccioni-Sohler M, Soares C, Papaiz-Alvarenga R, Castro M, Faria L, Peralta J. Neurologic dengue manifestations associated with intrathecal specific immune response. Neurology. 2009;73(17):1413-7.

23. Pan K, Roy U, Panwar A, Lal PK, Chakravarty S. Acute Disseminated Encephalomyelitis: A Rare Complication of Dengue Infection. Archives of Medicine. 2016.

24. Fragoso YD, Brooks JBB. Encephalomyelitis Associated With Dengue Fever. JAMA neurology. 2016;73(11):1368-.

25. Ferreira MLC, C. G.; Coelho, C. A.; Mesquita, S. D. [Neurological manifestations of dengue: study of 41 cases]. Arq Neuropsiquiatr. 2005;63(2b):488-93. Epub 2005/08/02. doi: /S0004-282x2005000300023. PubMed PMID: 16059604.

26. Wasay M, Channa R, Jumani M, Shabbir G, Azeemuddin M, Zafar A. Encephalitis and myelitis associated with dengue viral infection: Clinical and neuroimaging features. Clin Neurol Neurosurg. 2008;110(6):635-40.

27. Wasay MC, R.; Jumani, M.; Shabbir, G.; Azeemuddin, M.; Zafar, A. Encephalitis and myelitis associated with dengue viral infection. Clinical and neuroimaging features. Clinical Neurology and Neurosurgery. 2008;110(6):635-40. doi: 10.1016/j.clineuro.2008.03.011.
