# Supplementary material for: Post-dengue acute disseminated encephalomyelitis: A case report and meta-analysis
Source: PLoS Negl Trop Dis. 2017 Jun 30;11(6):e0005715. doi: 10.1371/journal.pntd.0005715 (PMC5509372; doi:10.1371/journal.pntd.0005715)
Supplement: S4 Table — (DOCX) [file pntd.0005715.s009.docx]

**S4 Table. Cerebrospinal fluid (CSF), magnetic resonance imaging (MRI) of brain and spinal cord results.**

| Author, year | MRI of SC | MRI of brain | CSF |
| --- | --- | --- | --- |
| Pal, 2016 [1] | HI intramedullary signal changes involving SC extending from C3 level downward until conus suggestive of myelitis with cord edema | Hyper-intensities involving bilateral periventricular and parietal region with signal changes involving right cerebellar peduncle with involvement of upper brainstem | Elevated CSF cell count, with predominant lymphocytosis, elevated CSF protein content, and normal glucose level. immunoglobulin synthesis index 0.72, immunoglobulin synthesis rate 6.5 mg/day with no oligo-clonal bands |
| Moura, 2004 [2] | ND | ND | ND |
| Abdulrazak, 2015 [3] | ND | Multiple areas of restricted diffusion of WM of the supratentorial compartment involving fronto parietal & temporal lobes & internal capsules | Negative |
| Sundaram, 2010 [4] | ND | Hypo-intense lesions on T1weighted images involving bilateral hippocampi, thalami, cerebellar hemispheres & posterior part of the pons. These lesions were HI on T2 weighted images | ND |
| Singh, 2015 [5] | Multifocal ill-defined bilateral supratentorial deep & subcortical WM HI with ill-defined cervical/proximal dorsal cord edema suggestive of ADEM | | Proteins 80 mg%, 75 cells /mm^3^ (predominantly lymphocytes) & normal sugar. |
| Gera, 2010, [6] | ND | Confluent HI on T2 weighted & T2 FLAIR images in the periventricular & subcortical WM bilaterally in the frontal & parietal lobes & also left temporal lobe with involvement of U fibers at some locations Symmetrical involvement of the thalami, midbrain, pons, middle cerebellar peduncle & the cerebellar hemispheres were also seen. T2- images showed small areas of hemorrhage involving both thalami & the cerebellum. The thalami, pons & cerebellar hemispheres showed restricted diffusion on DWI | Absent cells & mild rise in protein (58 mg/dl) & glucose (162 mg/dl) & negative PCR for HSV |
| Gupta, 2013 [7] | ND | T2- weighted hyper-intensities involving Rt temporal neocortex, cerebellum, B/L symmetrical periventricular & subcortical WM of frontal, parietal lobe & callosal–septal interface | 10 cells (all lymphocytes), mildly raised proteins (58 mg/dl) and normal glucose (68 mg%) |
| Yamamoto, 2002 [8] | Multiple high-intensity spotty lesions from Th-7 to Th-11 | No abnormal findings in the cerebrum & cerebellum | Mild elevation of protein (89mg/ml) & pleocytosis (21 cells/μl) |
| Kumar, 2014 [9] | ND | Subtle hyper-intensity in genu, splenium of corpus callosum & Rt centrum semiovale in T2 & FLAIR | Elevated protein 109 mg/dl with 30 cells (lymphocyte 60%) and N sugar (81 mg/dl) |
| Brito, 2007 [10] | ND | ND | ND |
| Bhat, 2010 [11] | ND | Symmetrical hyper-intensities on T2-weighted & FLAIR involving B/L cerebellar hemispheres, B/L middle cerebellar peduncles, brain stem, B/L thalami, hypothalamus & periventricular deep & subcortical WM | ND |
| Chakrabarti, 2015 [12] | ND | T1 hypo-intensities & T2/T2 FLAIR HI signal changes in both thalamic, parietal, & occipital WM, brainstem & corpus callosum | N sugar & cell count with raised protein (98mg/dl) |
| Cunha-matta, 2004 [13] | ND | T1 hypo & T2/FLAIR HI signal changes in the both thalamic, parietal WM & cerebellar region | N sugar & cell count with raised CSF protein (80mg/dl) |
| Chowdhury, 2011 [14] | Cervico-dorsal cord swelling & intra-substance long segment cord signal noted in the cervico-medullary region from C2 to D2 level. Irregular end plate changes with mild posterior bulge  at C5 - C6 levels also seen | Evidence of ill-defined focal T2 HI in the B/L fronto-parietal superficial & deep WM, cortical & Pontine swellings.  Ill-defined T2 & flair hypersensitive signal in the pons & extending into the right middle cerebral peduncle. | ND |
| Gupta, 2015 [15] | ND | ND | Clear fluid; M/E – no cells; proteins – 70 mg%, sugar – 45 mg%, CSF positive IgM. |
|  | ND | Large area of T1 & T2 hypo/ FlAIR HI in Rt BG, thalamus, cerebral peduncle with hemorrhagic foci, with similar areas in B/L frontal, Rt temporal, & B/L parietal WM predominantly in subcortical location | No cells & elevated protein (228 mg/dl) & normal glucose (75 mg/dl) |
| Dewan, 2016 [16] | ND | ND | ND |
| Gala, 2012 [17] | MRI of brain & cervical spine showed Multi-focal predominant WM lesions on T2-weighted images with involvement of thalamus | | ND |
| Verma, 2011[18] | A hypo-intensive lesion on a T1 image & a high intensity lesion on a T2 image, at the Th-7 to Th-9 levels of the SC | | N routine CSF analysis & anti-dengue IgM in CSF |
| de Sousa, 2006 [19] | ND | B/L hemorrhagic demyelination | ND |
| Koshy, 2012 [20] | ND | Demyelination along with intraocular, retro-bulbar & optic nerve head haematoma | ND |
|  | ND | Patchy areas of HI signals on FlAIR images | TLC 320/cmm with 85% lymphocytosis, CSF proteins 200 mg/dl. CSF glucose, chloride & adenosine deaminase were within N limits. |
| Karoli, 2016 [21] | T2 HI lesion at the dorsal SC level | 2 small T2 lesions on the brainstem | Anti-dengue IgM & IgG & increased protein |
| Puccioni-Sohler, 2009 [22] | ND | T2 HI signal changes in pons, adjacent medulla & cervical cord | A glucose of 70 mg/dl, protein was 92 mg/dl, cell count 6/mm^3^ with all lymphocytes |
| Pan, 2016 [23] | MRI of brain & SC Confirmed the presence of demyelination | | LC of 10/μl, a protein concentration of 36 mg/dl & a glucose level of 75mg/dl |
| Fragoso, 2016 [24] | ND | ND | ND |
| Ferreira, 2005 [25] | ND | ND | ND |
| Wasay, 2008 [26] | ND | ND | ND |
| Our present case | N | N | pleocytosis (61 cells/ml); elevation of protein, 1.68 g/l; glucose, 0.43 g/l; Chlor, 138 mmol/l; lactate 4.23, mmol/l |

Abbreviations; TLC= Total Leukocyte count, LC= Lymphocyte Count, CSF= Cerebrospinal fluid, HI= Hyper-Intense, FlAIR = fluid attenuated inversion recovery sequence, N= Normal, SC= Spinal Cord, WM= WM, ADEM= Acute Disseminated Encephalomyelitis.

**References**

1. Pal S, Sen K, Biswas NM, Ghosal A, Jaman SR, Kumar KY. Clinico-radiological profile and outcome of dengue patients with central nervous system manifestations: A case series in an Eastern India tertiary care hospital. J Neurosci Rural Pract. 2016;7(1):114.

2. Moura P, Cordeiro MT, Brito C, Lima Filho JLd, Arraes LC. Neurological aspects of the dengue infection in Brazilian patients during the DENV3 epidemicas. An Fac Med Univ Fed Pernamb. 2004;49(2):115-8.

3. Razak AN, N. K. Acute Demyelinating Encephalomyelitis in a Neonate Secondary to Dengue Infection. Indian Pediatr. 2015;52(6):534. Epub 2015/06/30. PubMed PMID: 26121739.

4. Sundaram CU, S. G.; Dakshinamurthy, K. V.; Borgahain, R. Acute disseminated encephalomyelitis following dengue hemorrhagic fever. Neurol India. 2010;58(4):599-601. Epub 2010/08/27. doi: 10.4103/0028-3886.68666. PubMed PMID: 20739802.

5. Singh GS, Gursharan; Arora, Sunita. Acute Disseminated Encephalomyelitis Following Dengue Infection. 2015.

6. Gera CG, U. Acute disseminating encephalomyelitis with hemorrhage following dengue. Neurol India. 2010;58(4):595-6. Epub 2010/08/27. doi: 10.4103/0028-3886.68661. PubMed PMID: 20739799.

7. Gupta M, Nayak R, Khwaja GA, Chowdhury D. Acute disseminated encephalomyelitis associated with dengue infection: a case report with literature review. J Neurol Sci. 2013;335(1):216-8.

8. Yamamoto Y, Takasaki T, Yamada K-i, Kimura M, Washizaki K, Yoshikawa K, et al. Acute disseminated encephalomyelitis following dengue fever. J Infect Chemother. 2002;8(2):175-7.

9. Sanjeev Kumar BN, S; Jayantee, K; Misra, UK. Acute Disseminated Encephalomyelitis following Dengue Virus Infection. J Neuroinfect Dis. 2014;5(139):2.

10. Brito CA, Sobreira S, Cordeiro MT, Lucena-Silva N. Acute disseminated encephalomyelitis in classic dengue. Rev Soc Bras Med Trop. 2007;40(2):236-8.

11. Bhat D. Acute disseminated encephalomyelitis: A rare central nervous system manifestation of dengue hemorrhagic fever. Journal of Pediatric Infectious Diseases. 2010;5(4):415-7.

12. Chakrabarti S. A case of acute disseminated encephalomyelitis following dengue infection. CHRISMED Journal of Health and Research. 2015;2(2):169.

13. Palma da Cunha-Matta ASM, S. A.; Cardoso de Almeida, A.; Aquilera de Freitas, V.; Carod Artal, F. J. Complicaciones neurológicas de la infección por el virus del dengue. Rev Neurol. 2004;39(3):233-7.

14. Chowdhury R, Siddiqui M, Mahbub M, Hasan O, Talukder A, Nabi S, et al. Dengue fever as a cause of acute disseminated encephalomyelitis (ADEM). J Med. 2011;12(2):185-7.

15. Gupta R, Gupta P, Sharma R. Dengue fever presenting as acute disseminated encephalomyelitis (ADEM). Journal, Indian Academy of Clinical Medicine. 2015;16(2):159.

16. Dewan R, Anuradha S, Sethi P, Ish P. Dengue presenting as hemorrhagic acute disseminated encephalomyelitis. MAMC Journal of Medical Sciences. 2016;2(1):54.

17. Gala HC, Avasthi BS, Lokeshwar MR. Dengue shock syndrome with two atypical complications. The Indian Journal of Pediatrics. 2012;79(3):386-8.

18. Verma R, Sharma P, Garg RK, Atam V, Singh MK, Mehrotra HS. Neurological complications of dengue fever: Experience from a tertiary center of north India. Annals of Indian Academy of Neurology. 2011;14(4):272.

19. de Sousa AM, Puccioni-Sohler M, Borges AD, Adorno LF, Alvarenga MP, Alvarenga RMP. Post-dengue neuromyelitis optica: case report of a Japanese-descendent Brazilian child. J Infect Chemother. 2006;12(6):396-8.

20. Koshy JM, Joseph DM, John M, Mani A, Malhotra N, Abraham GM, et al. Spectrum of neurological manifestations in dengue virus infection in Northwest India. Trop Doct. 2012;42(4):191-4.

21. Karoli R, Siddiqi Z, Fatima J, Maini S. Was it a case of acute disseminated encephalomyelitis? A rare association following dengue fever. J Neurosci Rural Pract. 2013;4(3):318.

22. Puccioni-Sohler M, Soares C, Papaiz-Alvarenga R, Castro M, Faria L, Peralta J. Neurologic dengue manifestations associated with intrathecal specific immune response. Neurology. 2009;73(17):1413-7.

23. Pan K, Roy U, Panwar A, Lal PK, Chakravarty S. Acute Disseminated Encephalomyelitis: A Rare Complication of Dengue Infection. Archives of Medicine. 2016.

24. Fragoso YD, Brooks JBB. Encephalomyelitis Associated With Dengue Fever. JAMA neurology. 2016;73(11):1368-.

25. Ferreira MLC, C. G.; Coelho, C. A.; Mesquita, S. D. [Neurological manifestations of dengue: study of 41 cases]. Arq Neuropsiquiatr. 2005;63(2b):488-93. Epub 2005/08/02. doi: /S0004-282x2005000300023. PubMed PMID: 16059604.

26. Wasay M, Channa R, Jumani M, Shabbir G, Azeemuddin M, Zafar A. Encephalitis and myelitis associated with dengue viral infection: Clinical and neuroimaging features. Clin Neurol Neurosurg. 2008;110(6):635-40.

27. Wasay MC, R.; Jumani, M.; Shabbir, G.; Azeemuddin, M.; Zafar, A. Encephalitis and myelitis associated with dengue viral infection. Clinical and neuroimaging features. Clinical Neurology and Neurosurgery. 2008;110(6):635-40. doi: 10.1016/j.clineuro.2008.03.011.
